# Study on the effects and mechanisms of different coal matrix structures on the desulfurization efficiency of Pseudomonas putida

**DOI:** 10.3389/fmicb.2026.1820915

**Published:** 2026-04-15

**Authors:** Chunming Ai, Jiazhen Cui, Pingping Sun, Chao Liu

**Affiliations:** 1 College of Safety Science and Engineering, Liaoning Technical University, Huludao, China; 2 Key Laboratory of Mine Thermal Disaster and Prevention, Ministry of Education, Huludao, China; 3 Tianjin Bohai Vocational Technical College, Tianjin, China; 4 Norin Mining Limited, Beijing, China

**Keywords:** biodegradation mechanism, coal desulfurization, FTIR, organic sulfur, pseudomonas putida, XPS

## Abstract

This study aims to reveal the influence mechanism of coal matrix structure on microbial desulfurization efficiency and clarify the regulatory effect of coal chemical structural characteristics on microbial desulfurization efficiency, providing theoretical support for the precise application of coal biodesulfurization technology. Pseudomonas putida was used as the functional strain for microbial desulfurization experiments on 092a and 100b coal samples with significant structural differences, and the characteristics of the desulfurized coal samples were characterized by X-ray photoelectron spectroscopy (XPS) and Fourier transform infrared spectroscopy (FTIR). The results showed that the organic sulfur removal rate of 100b coal reached 60.9%, which was much higher than the 17.6% of 092a coal; Pseudomonas putida could efficiently degrade various forms of organic sulfur such as thiophene, sulfide and sulfoxide in 100b coal, while only selectively removing sulfone-type sulfur in 092a coal, and FTIR characterization further confirmed that coal matrix characteristics are the core factor determining the desulfurization efficiency of this strain. The high aromaticity and high condensation degree of 092a coal resulted in significant steric hindrance of sulfur components, which limited the specific binding between enzymes and substrates, whereas the thiophene sulfur in 100b coal could be efficiently degraded through the 4S metabolic pathway, and this study clarifies the regulatory effect of coal chemical structural characteristics on microbial desulfurization efficiency, further supplementing the theoretical basis for the precise application of coal biodesulfurization technology.

## 1 Introduction

Coal is the basic energy and industrial raw material of China (Liu et al., 2021), and plays an important role in human life and production. The main utilization method of coal is direct combustion to obtain heat energy. If raw coal is directly burned without processing, it will cause serious environmental pollution, among which SO_2_ produced by combustion is the most harmful pollutant. This pollution problem did not attract worldwide attention until the 1950s (Channa et al., 2025). Therefore, it is crucial to reduce the sulfur content of coal before it is used as fuel. Sulfur compounds in coal exist in the form of inorganic sulfur and organic sulfur. Inorganic sulfur generally exists in coal as minerals such as pyrite, while organic sulfur mainly exists in the form of mercaptans, thiophenes, etc. (Dong et al., 2024; Marinov et al., 2009). Common pre-combustion desulfurization technologies for coal in China mainly include physical desulfurization, chemical desulfurization, microbial desulfurization, etc. (Wang et al., 2021; Zhang et al., 2005). Chemical desulfurization is carried out through redox reactions or acid-base neutralization reactions (Xia et al., 2022). Its advantage is that it can efficiently remove most inorganic sulfur and part of organic sulfur, but the reaction conditions are harsh, energy consumption is high, reaction products are difficult to recover, and secondary pollution is easy to occur (Zhao et al., 2018). Physical desulfurization adopts traditional mineral processing technologies such as gravity separation and magnetic separation, which is simple to operate, but it is only suitable for inorganic sulfur removal, and the desulfurization effect and coal recovery rate are significantly affected by the occurrence state of inorganic sulfur, and cannot treat organic sulfur. Common physical desulfurization methods include jigging desulfurization, magnetic separation desulfurization, and electrical separation desulfurization (Shi, 2011). Electrical separation desulfurization makes S-S bonds in coal break more easily than C-S bonds (Cheng et al., 2025). Biological desulfurization is carried out through the selective oxidation of microorganisms, which is environmentally friendly and economical. It cannot only accurately remove finely disseminated inorganic sulfur, but also degrade part of organic sulfur. With mild reaction conditions, low investment and operation costs, and little pollution, it is a green desulfurization technology with great development potential (Mohebali and Ball, 2016).

In recent years, many studies on microbial desulfurization have been carried out by scholars at home and abroad. Handayani et al. (2016) conducted desulfurization experiments using surface enzymatic activators, iron oxide, etc. The strain successfully removed organic sulfur and other sulfur forms from Tongdongcang coal by using MBT, indicating that the strain and its metabolites have a significant effect on reducing sulfur content (Handayani et al., 2016). Akimbekov et al. (2021) isolated Bacillus sp. RKB 7 from coal mine soil in Kazakhstan, confirming that it can achieve efficient biosolubilization of lignite under mild alkaline conditions, and revealed the structural characteristics of the product through multispectral and microscopic characterization, providing an experimental basis for the sustainable preparation of coal-based humic substances (Akimbekov et al., 2021). Zhang et al. (2008) applied Phanerochaete chrysosporium to experimental research on coal desulfurization, and investigated the optimal conditions for desulfurization by white-rot fungi with respect to coal particle size, coal slurry concentration, inoculum amount and culture time; under the optimal conditions, the total sulfur removal rate reached 35.77% (Zhang et al., 2008). Aytar et al. (2013) studied the desulfurization of Turkish coal using Trametes versicolor ATCC 200801 and Phanerochaete chrysosporium ME446. On the basis of optimizing several important influencing factors including pH, temperature, coal slurry concentration, desulfurization time and stirring speed, the microbial desulfurization rate reached 40% (Aytar et al., 2013). Oluwafolakemi and Olawale (2023) used Pseudomonas putida for diesel desulfurization, and the total sulfur removal rate of diesel was 50.02%. The core pathways of microbial desulfurization mainly include sulfur oxidation, C-C bond cleavage and C-S bond cleavage (Gupta et al., 2004). The removal mechanism of inorganic sulfur is that microorganisms oxidize pyrite in the presence of oxygen and water to produce sulfate radicals and Fe2+, releasing heat (Liu et al., 2022; Soleimani et al., 2007). The removal mechanism of organic sulfur generally includes two pathways, namely the Kodama pathway via C-C bond cleavage and the 4S metabolic pathway via specific C-S bond cleavage (Tong et al., 2005). Microorganisms can grow in a sulfur-containing coal environment (Akimbekov et al., 2022; Guan et al., 2020; Pokorný et al., 2005), and bacterial solubilization of coal can promote microbial activity (Altynbay et al., 2024), and these characteristics can effectively improve the efficiency of biological desulfurization.

This study selected Pseudomonas putida as the experimental strain to conduct desulfurization experiments on 092a coal and 100b coal from different mining areas in Datong, Shanxi. It aims to investigate the mechanism by which Pseudomonas putida achieves efficient desulfurization in the more responsive coal. Fourier transform infrared spectroscopy (FTIR) and X-ray photoelectron spectroscopy (XPS) were employed to characterize the raw coal and solid-phase products after desulfurization, in order to deeply analyze the microscopic mechanisms of microbial desulfurization.

## 2 Experimental section

### 2.1 Experimental materials and preparation

The experimental coal samples selected were 092a coal and 100b coal from the Datong mining area in Shanxi Province. After natural drying, crushing and uniform mixing, the coal samples were all sieved through a 0.20 mm sieve in accordance with national standards (Test Sieves, 2000), followed by sterilization at 180 °C for 2 h. The forms of sulfur in the coal samples used in the experiment were analyzed according to GB/T 215-2003 (National Technical Committee of Coal Standardization, 2003), and the model of the coulometric sulfur detector used was Sunde SDS616. The analysis results of the forms of sulfur in the two coal samples are shown in Table 1. NB liquid medium formulation: peptone 5 g, beef extract powder 3 g, NaCl 5 g, distilled water 1,000 mL, pH 7.0.

**TABLE 1:** Forms of sulfur in two coal samples before desulfurization.

Coal sample	Total sulfur St,d (%)	Total sulfur St,d (%)	Pyritic sulfur Sp,d (%)	Organic sulfur So,d (%)
092a	4.03	0.02	0.66	3.35
100b	3.05	0.08	0.79	2.18

### 2.2 Strain culture

Pseudomonas putida was purchased from Shanghai Chunshi Biotechnology Co., Ltd., with the strain number CS-K401J = ATCC49128. The strain was cultured in NB liquid medium. It was activated according to the culture method provided by the supplier, and reserved for later use after 3 successive subcultures.

**FIGURE 1:**
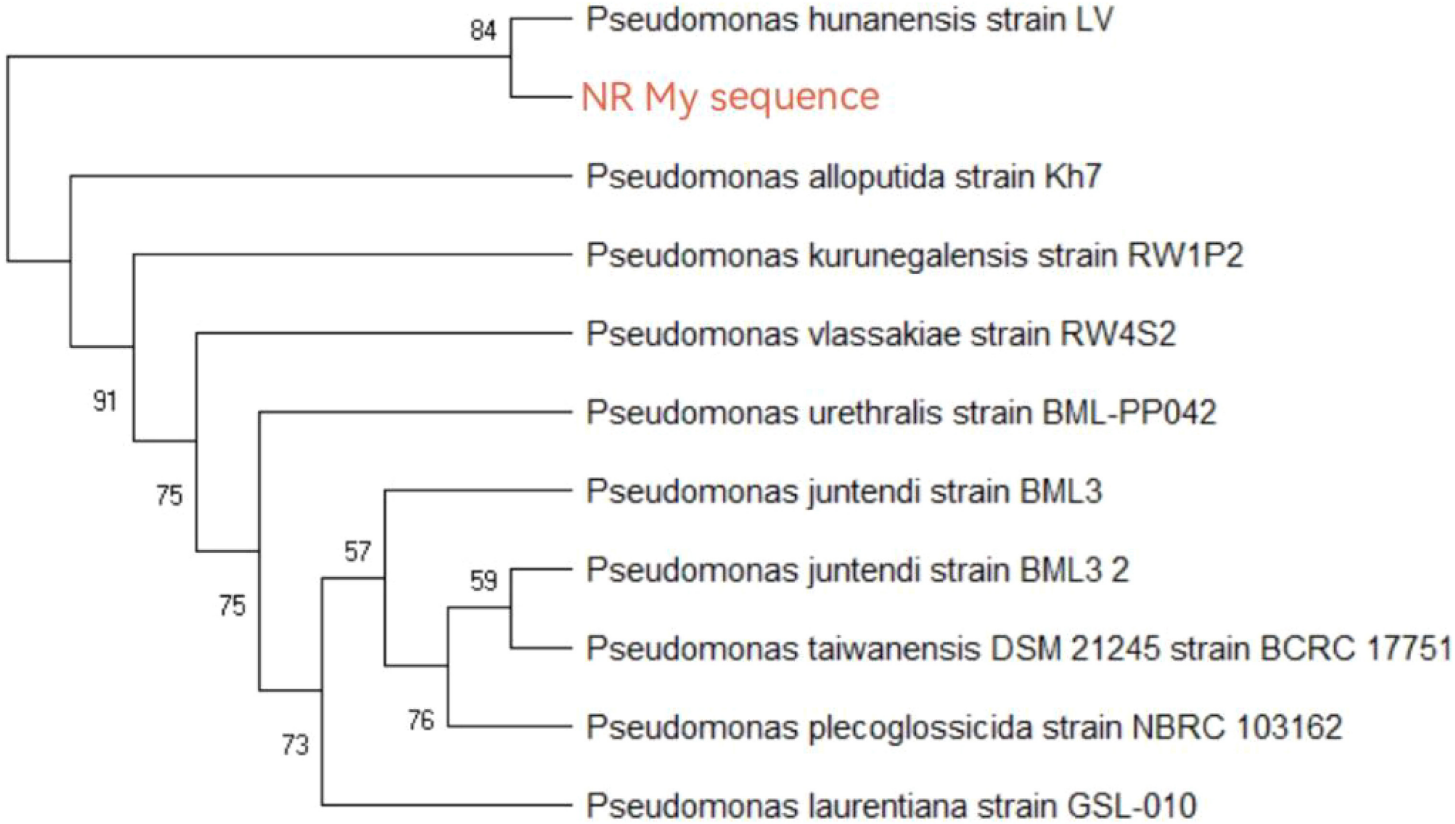
Phylogenetic tree of 16S rRNA.

To ensure the strain was in a good active state to meet the requirements of subsequent desulfurization experiments, 16S rRNA purification and sequencing were performed on the selected strain to confirm its taxonomic status. The obtained 16S rRNA sequence was submitted to the GenBank database, and homology analysis was conducted with related sequences using BLAST. Sequence comparison results showed that the homology between this strain and multiple Pseudomonas putida strains reached more than 99%. To further verify the phylogenetic relationship of the strain, the 16S rRNA sequences of 10 reference strains with high homology to this strain were selected, and a phylogenetic tree was constructed using MEGA 10 software, as shown in Figure 1. The analysis results further confirmed that the strain used in this study was Pseudomonas putida.

The microbial desulfurization process was carried out in 500 mL conical flasks. The well-cultured Pseudomonas putida strain was inoculated into 200 mL of NB liquid medium, and cultured in a constant-temperature shaking incubator at 30°C with a rotation speed of 30 r/min for 2 days.

### 2.3 Microbial desulfurization experiment of coal

**FIGURE 2:**
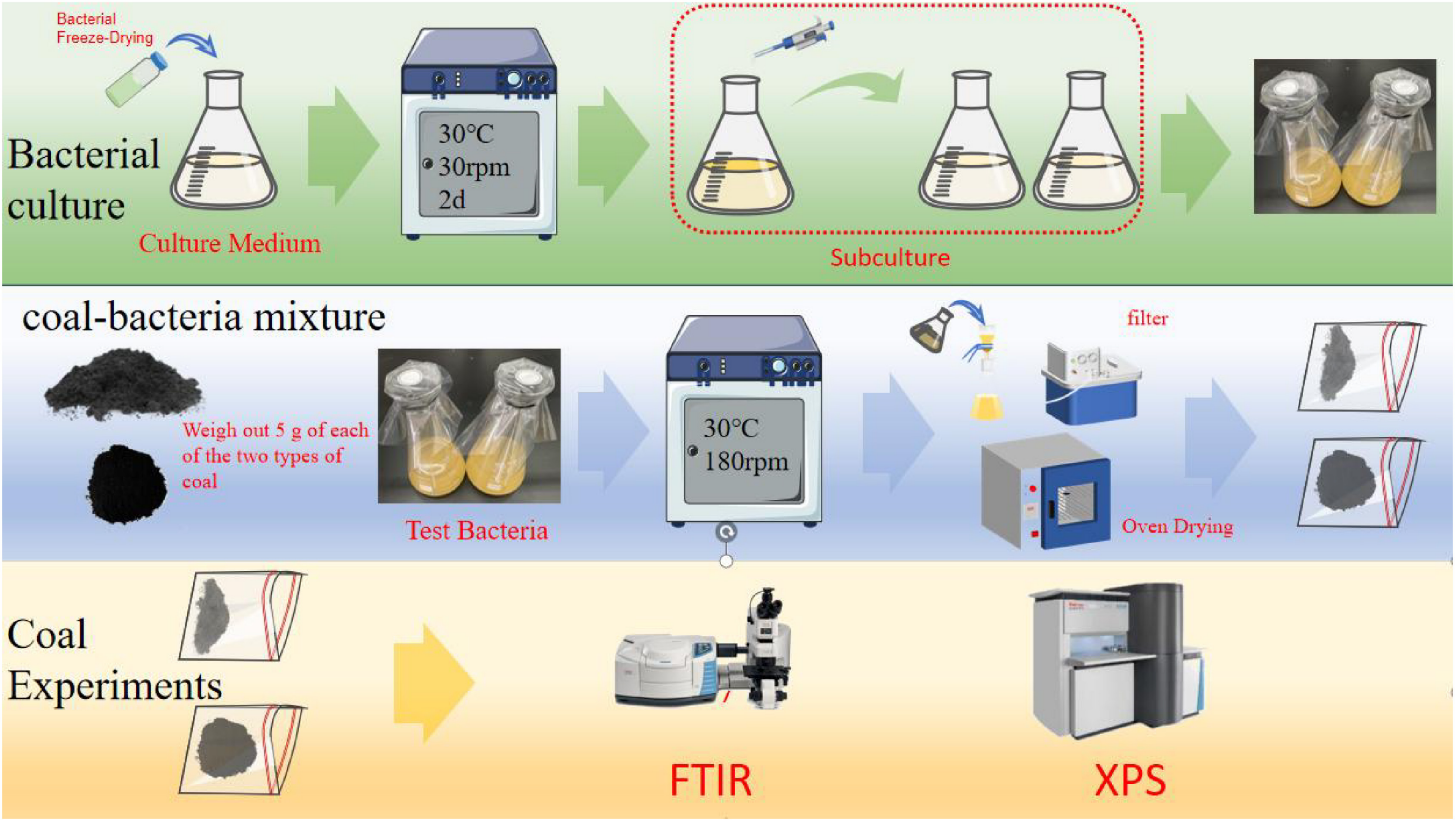
Experimental flow chart.

5 g of each coal sample was weighed and added into Erlenmeyer flasks containing NB medium, sterilized in a vertical pressure steam sterilizer (121°C, 20 min), and then cooled in a single-person single-sided clean bench (SW-CJ-1FD). After cooling, bacterial suspension was inoculated into each Erlenmeyer flask, followed by incubation in a constant temperature shaker for 24 h. The coal samples were then filtered and collected, and washed repeatedly with deionized water until no residual microorganisms were detected. The two coal samples were dried in an oven at 80°C for 48 h. Finally, the morphological sulfur analysis of the coal samples was carried out. The experimental flow chart is shown in Figure 2.

### 2.4 Physicochemical properties analysis of coal

X-ray photoelectron spectroscopy (XPS) measurements were performed on the two coal samples before and after microbial desulfurization using a Thermo Scientific K-ALPHA X-ray photoelectron spectrometer. The purpose was to analyze the structure of organic compounds in the coal matrix and detect changes in the content of various organic sulfur forms. The analysis chamber was evacuated to a vacuum of 10–8 Pa or lower. The X-ray excitation source power was 150 W, and the analysis area was 300 × 700 μm. The binding energy in the full-spectrum energy analysis was calibrated.

Fourier transform infrared spectroscopy (FTIR) tests were conducted on the coal samples before and after microbial desulfurization using a BRUKER Fourier transform infrared spectrometer. The tests aimed to analyze the functional group structure of organic compounds in the coal samples and detect changes in sulfur-containing functional groups. The coal samples were ground, dried, and mixed with KBr at a mass ratio of 1:200 to prepare pellets. The samples were scanned 32 times at room temperature with a spectral resolution of 4.0 cm^−1^, covering a spectral range of 400–4,000 cm^−1^.

## 3 Results and discussion

### 3.1 Changes in sulfur forms of two coal samples before and after desulfurization

**FIGURE 3:**
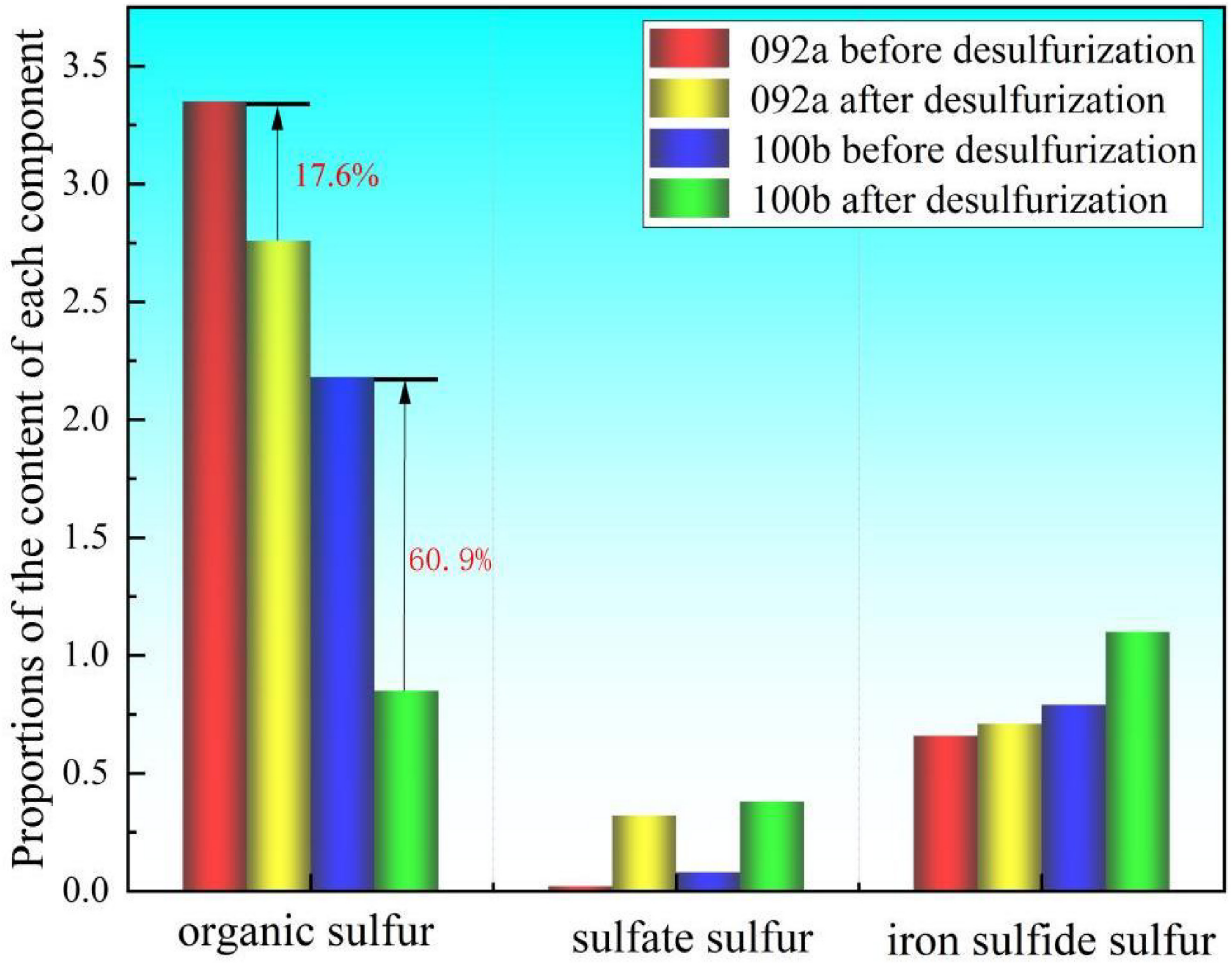
Forms of sulfur in two coal samples before and after desulfurization.

According to the data in Figure 3, the organic sulfur content of both coal samples showed a decreasing trend after desulfurization. Among them, the organic sulfur removal rate of 100b coal reached 60.9%, which confirms the efficient degradation capacity of Pseudomonas putida for organic sulfur. It also indicates that the occurrence form of organic sulfur in 100b coal is more easily recognized and degraded by the enzyme system of the strain. In contrast, the organic sulfur removal rate of 092a coal was only 17.6%, much lower than that of 100b coal, showing a significant difference in desulfurization efficiency between the two. Under the same experimental conditions, the two coal samples with obvious differences in structural characteristics exhibited drastically different desulfurization effects. Thus, it can be inferred that coal matrix structure is a key factor determining microbial desulfurization efficiency.

The sulfate sulfur content of both coal samples increased significantly, which is direct evidence of microbial oxidative desulfurization. During the desulfurization process, Pseudomonas putida performs oxidative metabolism on both organic sulfur and inorganic sulfur in coal, ultimately converting them into soluble sulfate ions. Some of these sulfate ions are adsorbed on the surface of coal samples, leading to an increase in the detected sulfate sulfur content.

The pyritic sulfur content of both coal samples showed an increasing trend, which is not due to an increase in the absolute content of pyrite but a change in its relative proportion. During the microbial desulfurization process, a large amount of organic sulfur is removed, leading to a reduction in the total mass of the coal samples. However, pyrite is difficult to be degraded by this strain due to its stable structure, resulting in minimal loss. Consequently, its relative percentage in the remaining coal samples increases instead. In summary, Pseudomonas putida has limited ability to remove pyritic sulfur, and its desulfurization target is mainly focused on organic sulfur.

### 3.2 Changes in chemical composition of two coal samples before and after desulfurization

**FIGURE 4:**
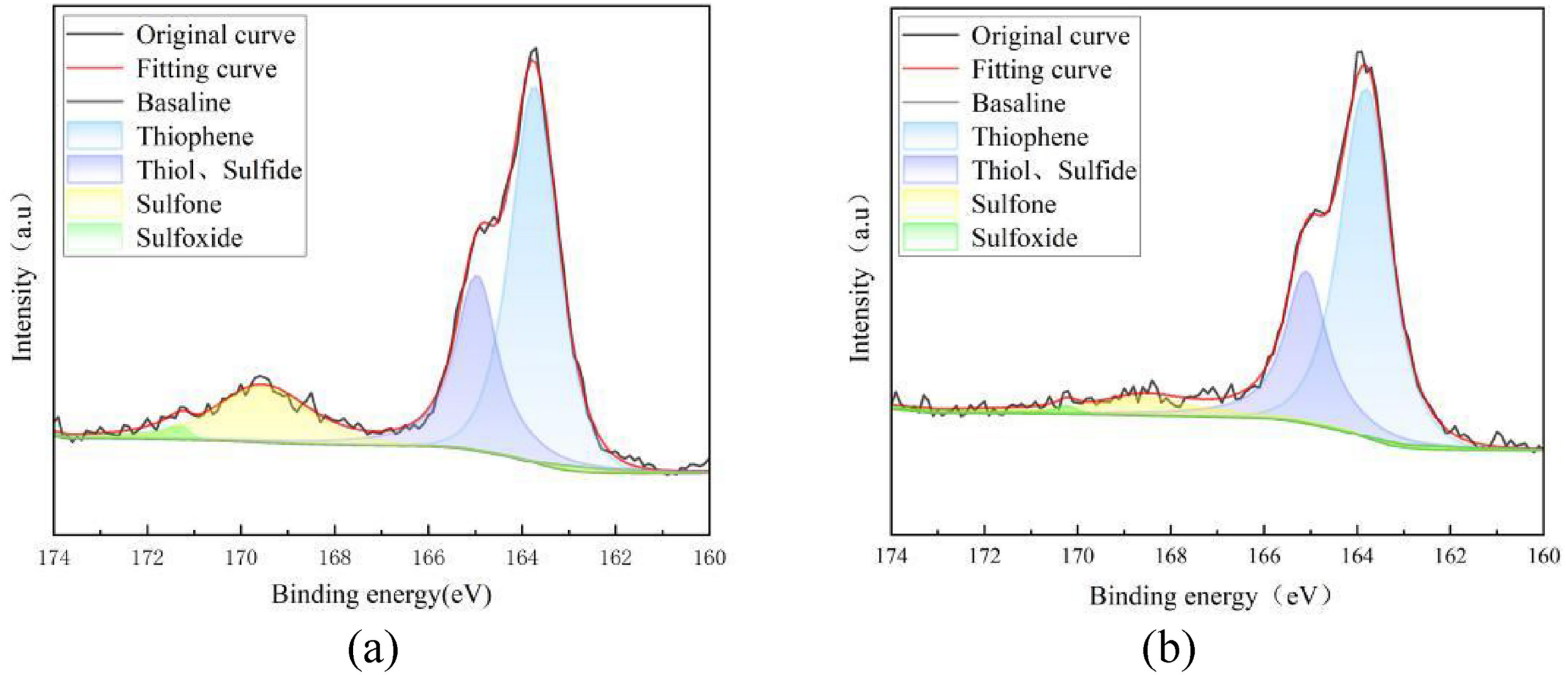
Peak-differentiating and fitting of XPS-S2P spectrum of 092a coal. **(a)** Fitting results of 092a before desulfurization. **(b)** Fitting results of 092a after desulfurization.

**FIGURE 5:**
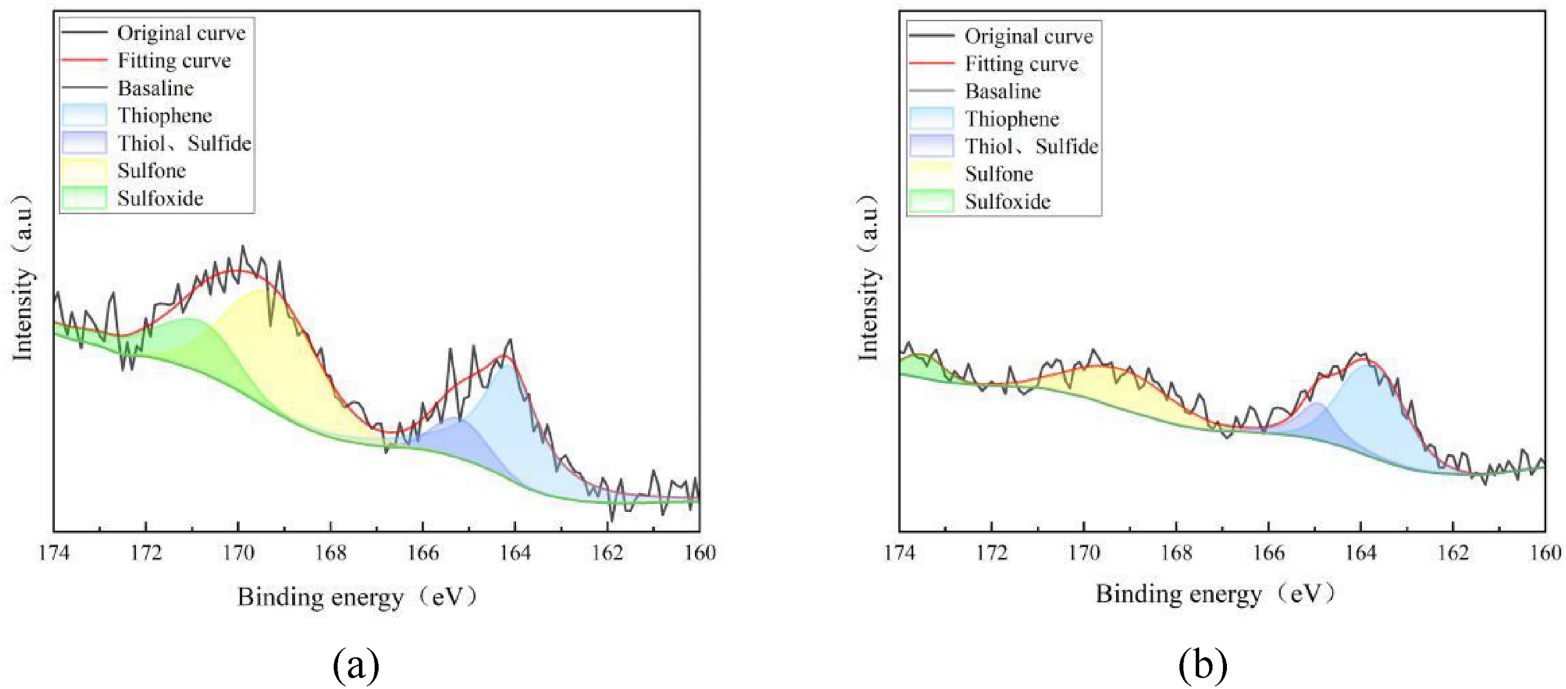
Peak-differentiating and fitting of XPS-S2P spectrum of 100b coal. **(a)** Fitting results of 100b before desulfurization. **(b)** Fitting results of 100b after desulfurization.

Combined with the results of sulfur form analysis in the previous section, there is a significant difference in the organic sulfur removal rates between 092a coal and 100b coal. To clarify the essential reason for this difference, X-ray photoelectron spectroscopy (XPS) was used to perform peak fitting analysis on the S2p spectra of the two coal samples before and after desulfurization, aiming to explore the differences in organic sulfur forms and desulfurization selectivity. The results are shown in Figures 4, 5.

As can be seen from Figure 4, there is no significant change in the fitted peak intensities of thiophene, thiol and sulfide, and only the fitted peak intensity of sulfone shows a significant decrease. This indicates that Pseudomonas putida exhibits an obvious removal effect on the sulfone-form organic sulfur in 092a coal, while it has no obvious removal effect on organic sulfur in forms such as thiophene, thiol, sulfide and sulfoxide.

As can be seen from Figure 5, the fitted peak areas of thiophene, thiol and sulfide, sulfone, and sulfoxide all show a downward trend after desulfurization of 100b coal. This indicates that Pseudomonas putida exerts a removal effect on organic sulfur in various forms in 100b coal, which is consistent with the higher organic sulfur removal rate of 100b coal observed in the previous analysis of sulfur forms.

The XPS results indicate that Pseudomonas putida exhibits differences in the selectivity of organic sulfur removal between the two coals, essentially due to the varying enzyme-substrate compatibility caused by the characteristics of the coal matrix. The bacterium shows significant removal capacity for sulfone-type sulfur in both coals, demonstrating that the secreted enzyme system possesses high catalytic activity and substrate specificity toward sulfone structures. However, there are obvious differences in the removal effects of organic sulfur in forms such as thiophene, thiol, sulfide, and sulfoxide, which directly leads to a marked disparity in the desulfurization rates of the two coals. Among them, the removal effect of thiophene-type organic sulfur in 092a coal is unsatisfactory, indicating that the removal of this type of sulfur form requires matching with specific compatible strains. In contrast, the efficient removal of various organic sulfur forms in 100b coal precisely explains its higher desulfurization efficiency. In summary, the difference in removal selectivity between the two coals directly proves that the sulfur form distribution in the coal matrix is the main factor determining the selectivity of microbial desulfurization, and also the key reason for the desulfurization rate difference mentioned earlier.

**FIGURE 6:**
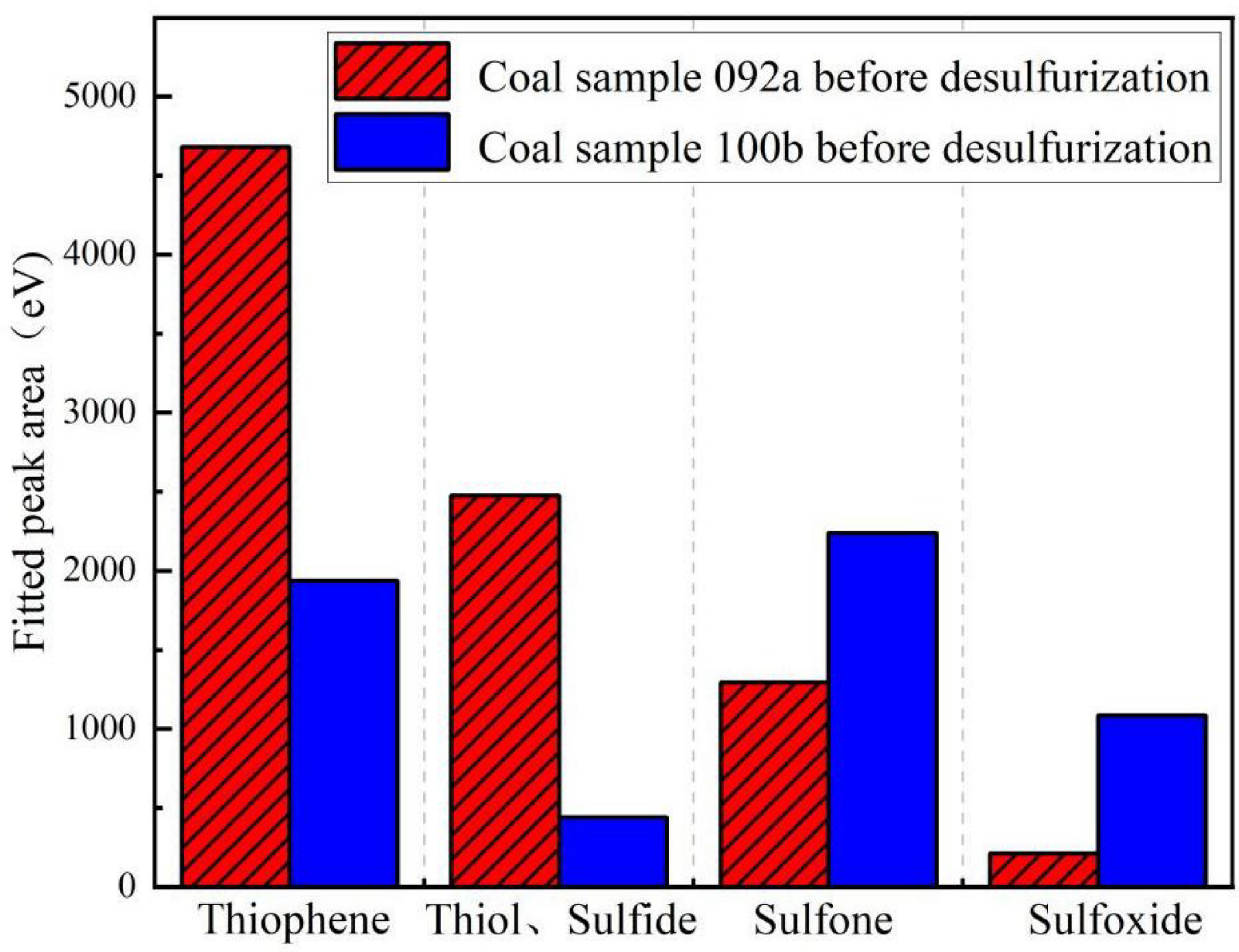
Fitted peak areas of each component in the two coals before desulfurization.

Based on the fitted peak area data obtained from X-ray photoelectron spectroscopy (XPS), the relative content proportions of various organic sulfur species were calculated using the peak area normalization method. This approach clearly quantifies the magnitude of changes in each organic sulfur fraction for different coal samples before and after biological desulfurization. Figure 6 shows the contents of four forms of organic sulfur (thiophene, mercaptans and sulfides, sulfones, sulfoxides) in coal samples 092a and 100b before desulfurization. The results indicate that the contents of sulfones and sulfoxides in the 092a coal matrix are lower than those in 100b coal, while the contents of thiophene, mercaptans and sulfides are higher. It can be concluded that organic sulfur related to aromatic rings, aliphatic sulfur bridges and side chain structures accounts for a higher proportion in 092a coal. Since aromatic ring structures are more stable than sulfone (R-SO_2_-R) structures and harder to be degraded by microbial enzyme systems, this also exacerbates the differences in desulfurization efficiency between the two coal samples, which is logically consistent with the previous morphological sulfur analysis results.

**FIGURE 7:**
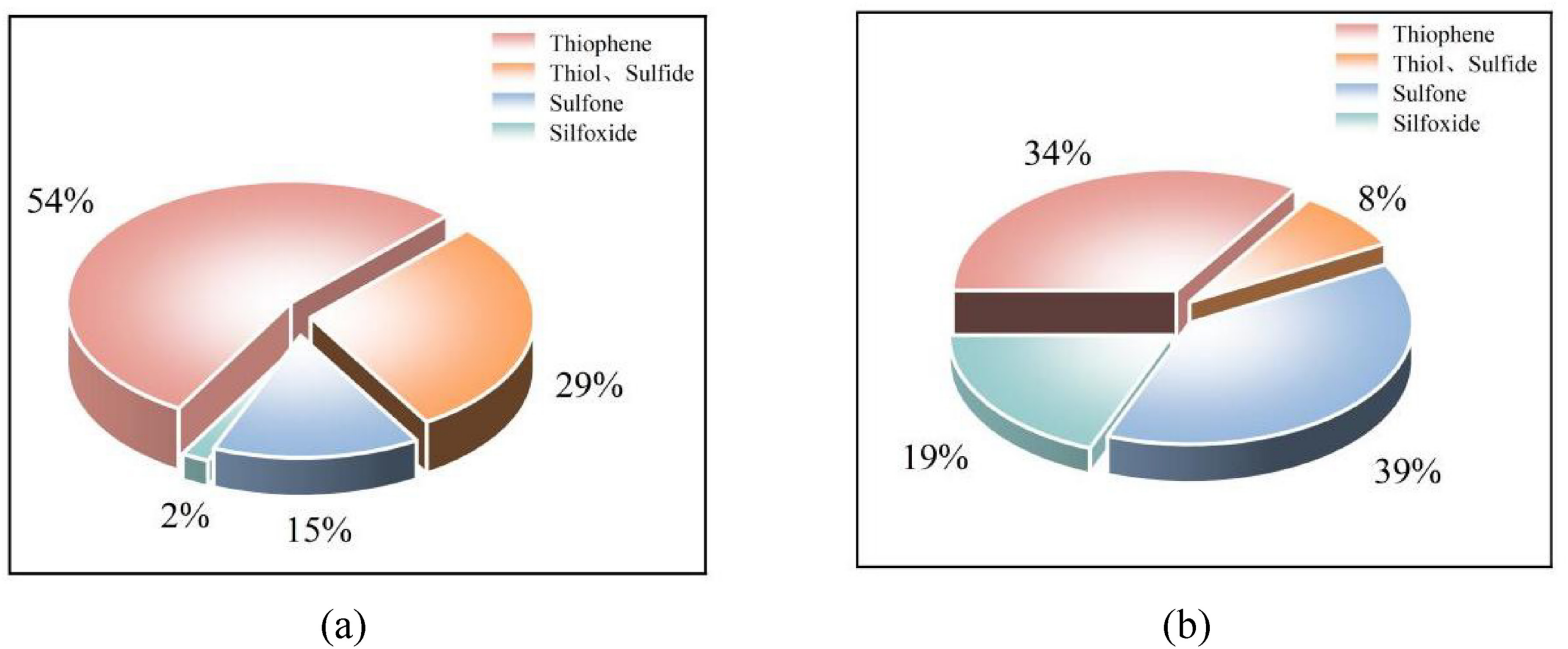
Proportions of existing forms of organic sulfur in the two coals. **(a)** Proportion of existing forms of organic sulfur in coal sample 092a. **(b)** Proportion of existing forms of organic sulfur in coal sample 100b.

As shown in Figure 7, the organic sulfur in coal sample 092a mainly exists in the form of thiophene. It is inferred that the 092a coal matrix is dominated by condensed aromatic ring structures, containing only a small amount of aliphatic sulfur bridges and side chains. The organic sulfur in coal sample 100b is mainly present as sulfones and thiophenes, with its matrix structure dominated by aromatic rings and sulfone structures. The higher aromaticity and condensation degree of 092a coal result in thiophene and sulfide-type organic sulfur mostly occurring inside the condensed ring structures. Significant steric hindrance hinders the effective binding between enzymes and substrates, which is also an important reason for its organic sulfur removal rate of only 17.6%. In contrast, the structure of 100b coal is relatively open, with higher contents of aliphatic components and small aromatic clusters. Its sulfur components have good exposure, making them more easily recognized and acted upon by microbial enzyme systems, ultimately achieving an efficient removal rate of 60.9%. In addition, the sulfone-specific degrading enzymes secreted by the strain can function efficiently in both coal samples, indicating that the catalytic mechanism of this enzyme for sulfone structures has good universality.

### 3.3 Changes in functional groups of the two coals before and after desulfurization

**FIGURE 8:**
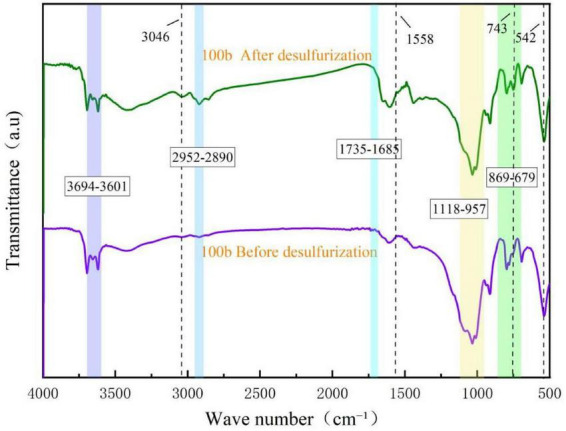
FT-IR spectra of Coal 092a before and after desulfurization.

**FIGURE 9:**
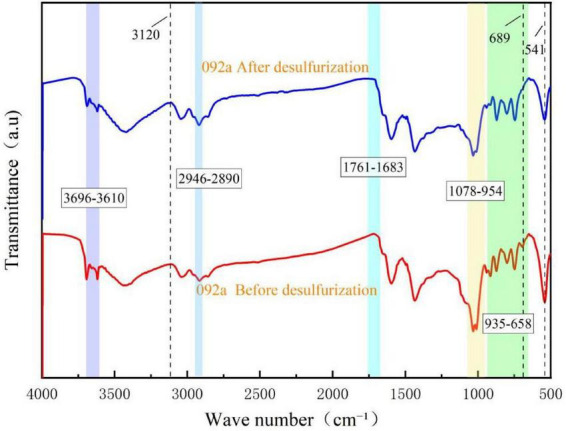
FT-IR spectra of Coal 100b before and after desulfurization.

To reveal the differences in the impact of coal matrix structure and changes in functional groups on desulfurization efficiency, Fourier transform infrared (FTIR) spectroscopy analysis was performed on two types of coal before and after desulfurization, and the results are shown in Figures 8, 9.

As can be seen from Figure 8, the infrared spectrum of the coal sample after desulfurization did not undergo an overall change compared with the raw coal, with differences only in the intensity or position of some spectral peaks. The wavenumber range of 3,696–3,610 cm^–1^ corresponds to the stretching vibration peak of hydroxyl groups (-OH). The decreased intensity of this peak after desulfurization is speculated to result from the reduced hydrogen bonding with hydroxyl groups following the removal of sulfides. The peak at 3,120 cm^–1^ is the stretching vibration peak of aromatic C-H bonds; the intensity change before and after desulfurization indicates the stability of the aromatic ring structure and a decrease in the content of thiophene rings. The wavenumber range of 2,946–2,890 cm^–1^ corresponds to the vibration peak of aliphatic -CH_2_- bonds, and the change before and after desulfurization reflects the cleavage and oxidation reactions of aliphatic chains. The wavenumber range of 1,761–1,683 cm^–1^ belongs to the oxygen-containing functional group region, where the vibration is attributed to the stretching vibration of C = O in carboxylic acids or ketones. The decreased intensity after desulfurization may be due to the removal of sulfonic acid groups. The presence of C-O or S = O stretching vibrations near the wavenumber range of 1,078–954 cm^–1^ indicates a reduction in the number of sulfate esters or ether bonds after desulfurization. Within the wavenumber range of 658–935 cm^–1^, the disappearance of the characteristic peak at 689 cm^–1^ suggests the cleavage of C-S bonds, with sulfur removed from the aromatic rings by converting thiophene sulfur into SO_4_^2–^ through the 4S metabolic pathway. The vibration at 831 cm^–1^ indicates that microorganisms desulfurize via oxidation pathways, possibly generating sulfoxide or sulfone intermediates. The decreased intensity of all characteristic peaks in the wavenumber range of 750–871 cm^–1^ indicates that the microbial attack on aromatic rings caused changes in their electron density. In addition, the peak intensity at 541 cm^–1^ decreased; this characteristic peak is related to the vibration mode of minerals in the coal sample, reflecting that microorganisms exerted a certain influence on the structure of inorganic minerals in coal during the desulfurization process. Sulfur in coal 092a mainly exists in the form of sulfonic acid groups and thiophene, so the removal of sulfur had a significant impact on oxygen-containing functional groups and aromatic ring substitution patterns.

As can be seen from Figure 9, compared with the infrared spectrum of raw coal, the infrared spectrum of the coal sample after desulfurization did not undergo significant changes, with differences only at some spectral peaks. The wavenumber range of approximately 3,694–3,601 cm^–1^ corresponds to the stretching vibration peak of hydroxyl groups (-OH). The decreased peak intensity after desulfurization indicates that hydroxyl-containing sulfides were degraded during the microbial desulfurization process. The characteristic vibration peak of aliphatic hydrocarbons is located at 2,952–2,890 cm^–1^; the reduced peak intensity suggests that microorganisms degraded aliphatic chains or cleaved thioether bonds. The wavenumber range of 1,735–1,685 cm^–1^ belongs to the vibration region of oxygen-containing functional groups, and the decreased peak intensity indicates that sulfur-containing carbonyl compounds were metabolized by microorganisms. The stretching vibration of aromatic ring skeleton C = C exists near 1,558 cm^–1^, indicating that microorganisms altered the content and state of these functional groups through metabolic activities. The presence of C-O or S = O stretching vibrations near 1,118–975 cm^–1^ indicates a reduction in the number of sulfate esters or ether bonds after desulfurization. The stretching vibration peak of aromatic ring C-H is located near 3,046 cm^–1^, suggesting that bacteria attacked thiophene heterocycles in coal during desulfurization. The stretching vibrations in the wavenumber range of 869–679 cm^–1^ correspond to the out-of-plane bending vibration of C-H in aromatic structures, and also reflect the degradation behavior of sulfur-containing heterocycles. The disappearance of the characteristic peak at 743 cm^–1^ indicates the cleavage of C-S bonds. The vibration at 832 cm^–1^ indicates that the desulfurization process is oxidative desulfurization, where dibenzothiophene (DBT) is converted to dibenzothiophene sulfone (DBTO_2_). The peak intensity at 542 cm^–1^ decreased; this characteristic peak is related to the vibration mode of minerals in the coal sample, reflecting that microorganisms exerted a certain influence on the structure of inorganic minerals in coal during the desulfurization process.

FT-IR spectroscopy analysis shows that Pseudomonas putida possesses cometabolic capacity for aromatic compounds (Tsugawa et al., 2019) and can degrade organic substances such as dibenzothiophene (DBT). To quantitatively evaluate the impact of coal molecular structure on desulfurization efficiency, the characteristic absorption peaks of coal samples can be divided into four categories: hydroxyl groups (3,000–3,700 cm^–1^), aliphatic groups (3,000–2,800 cm^–1^), oxygen-containing functional groups (1,800–900 cm^–1^), and aromatic hydrocarbons (900–700 cm^–1^), and peak fitting and analysis were performed for these four wavebands.

#### 3.3.1 Peak fitting of the hydroxyl group region

**FIGURE 10:**
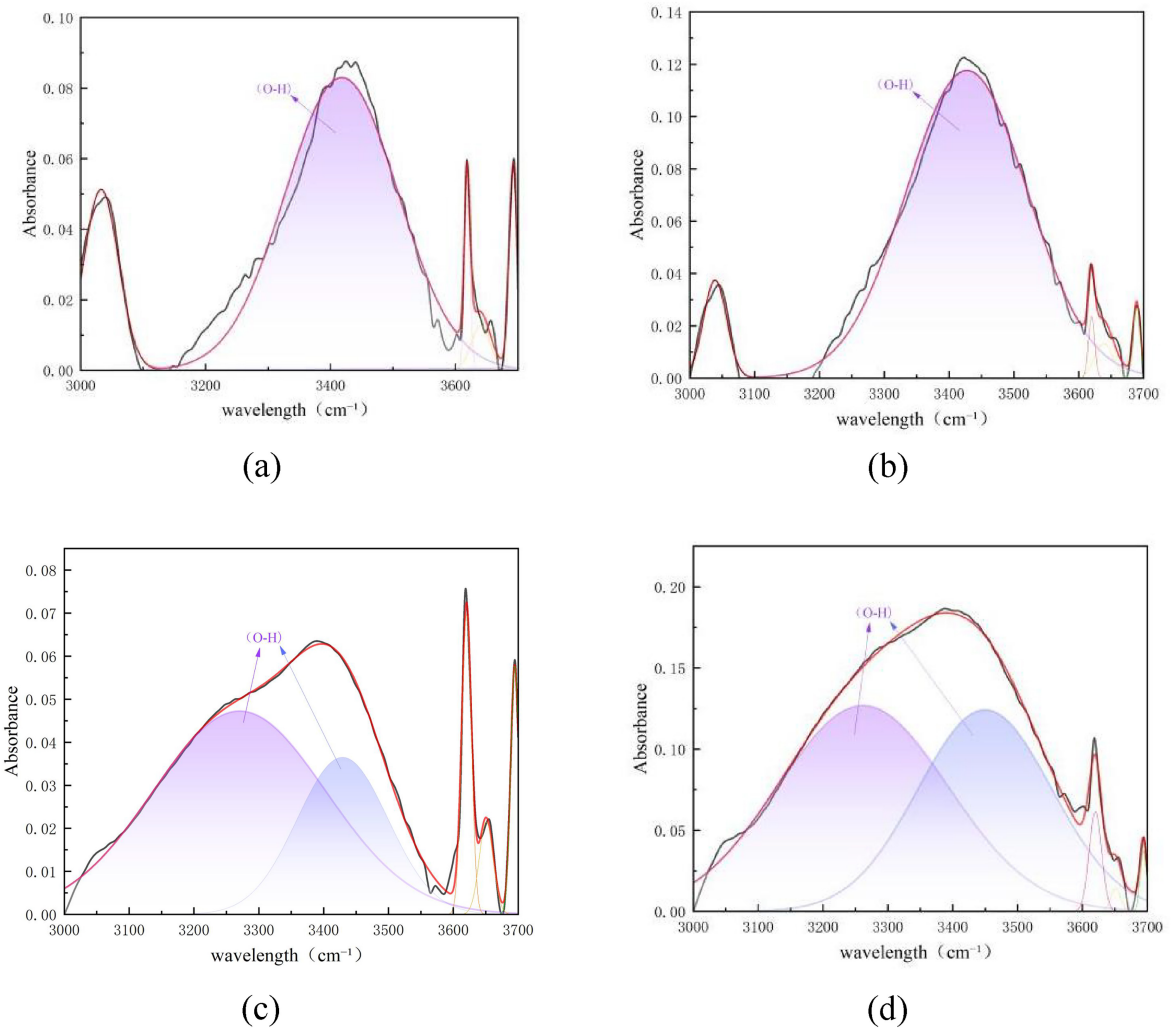
Peak fitting of the two types of coal in the hydroxyl group region before and after desulfurization. **(a)** Fitting results of Coal 092a before desulfurization. **(b)** Fitting results of Coal 092a after desulfurization. **(c)** Fitting results of Coal 100b before desulfurization. **(d)** Fitting results of Coal 100b after desulfurization.

**FIGURE 11:**
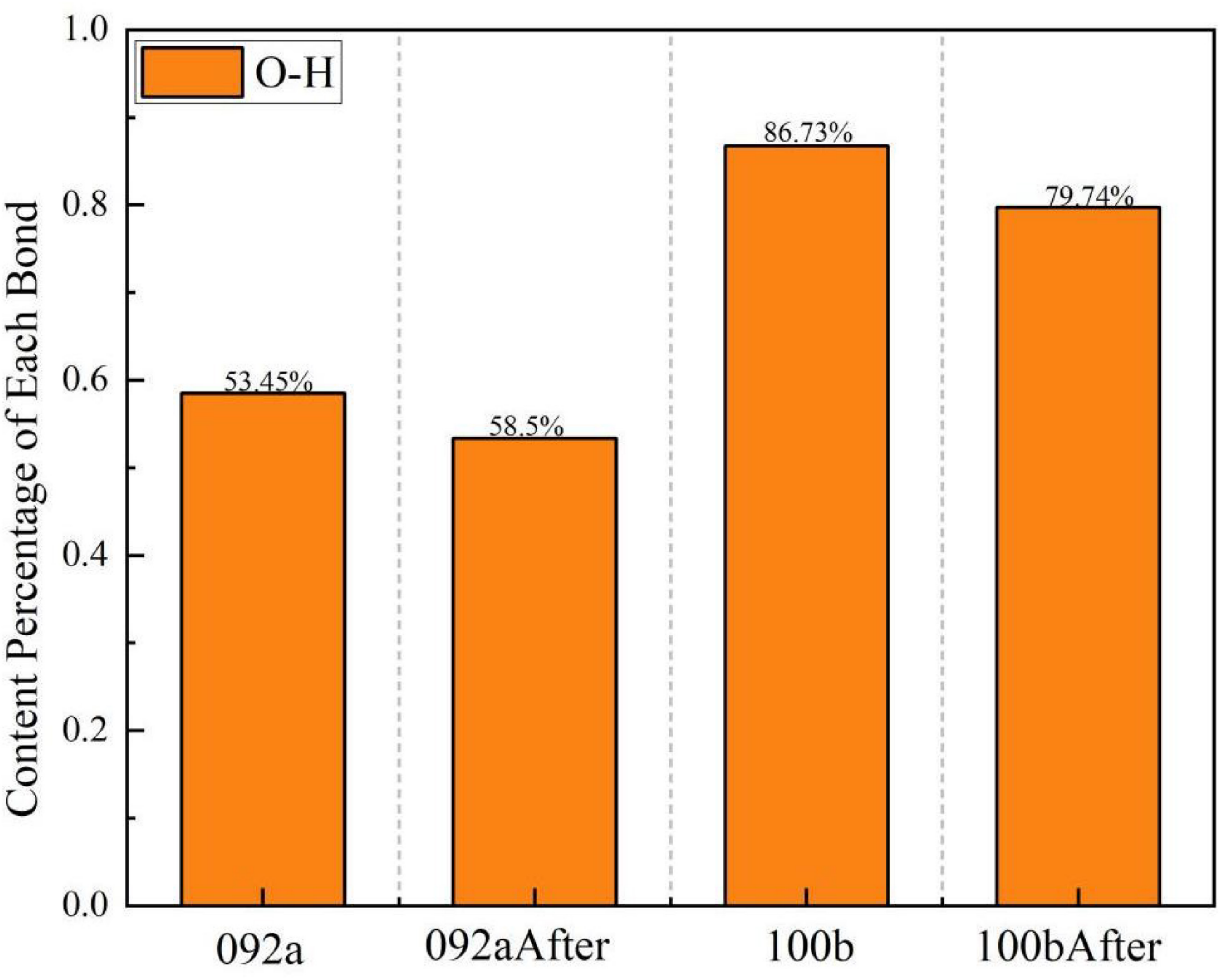
Content proportions of each component in the hydroxyl group region.

Figure 10 shows the peak fitting results of the two types of coal in the hydroxyl group region (3,000–3,500 cm^–1^) before and after desulfurization. The content proportion of each component in this region was calculated based on the fitted peak area in Figure 10, as presented in Figure 11.

The desulfurization process of Pseudomonas putida significantly affects the hydrogen bond network in the coal molecular structure, and this effect varies between Coal 092a and Coal 100b. Such differences reveal the interaction mechanism between the desulfurization process and the chemical structure of the coal matrix.

As can be seen from Figure 11, the fitting area of O-H bonds in Coal 092a decreased from 58.5 to 53.45%, and that in Coal 100b dropped from 86.73 to 79.74%. After microbial desulfurization treatment, the hydrogen bonding interactions related to hydroxyl groups in both types of coal were weakened, with a more significant impact on Coal 100b. The core reason for the difference lies in the different contents of matrix hydrogen bond acceptors in the two types of coal. Coal 100b contained more sulfur-containing functional groups before desulfurization, which could form more abundant hydrogen bonds with hydroxyl groups. After desulfurization, a large number of sulfur-containing functional groups were removed, leading to a reduction in hydrogen bond acceptors. Consequently, the hydrogen bonding interactions related to hydroxyl groups were significantly weakened, resulting in a more obvious decrease in the proportion of O-H bonds. In contrast, most of the sulfur-containing functional groups in Coal 092a are embedded in condensed ring structures, and their hydrogen bonding interactions with hydroxyl groups were inherently weak. Therefore, the changes in the hydroxyl group region were more moderate after desulfurization.

In addition, during the metabolic process of microorganisms, in addition to specifically attacking C-S bonds, they may also oxidize and cleave some aliphatic side chains, ether bonds, and weak aromatic structures in coal through cometabolic or non-specific actions. This degradation effect changes the chemical environment of coal molecules, further affects the hydrogen bonding state of hydroxyl groups, and ultimately leads to a reduction in the proportion of the fitting peak area of O-H bonds.

The difference in the degree of decrease in O-H bond content between the two types of coal confirms that desulfurization efficiency is closely related to the chemical structure of the coal matrix. Among them, Coal 100b, with a looser structure and more abundant oxygen-containing functional groups, is more significantly affected by the desulfurization process.

#### 3.3.2 Peak fitting of the aliphatic region

**FIGURE 12:**
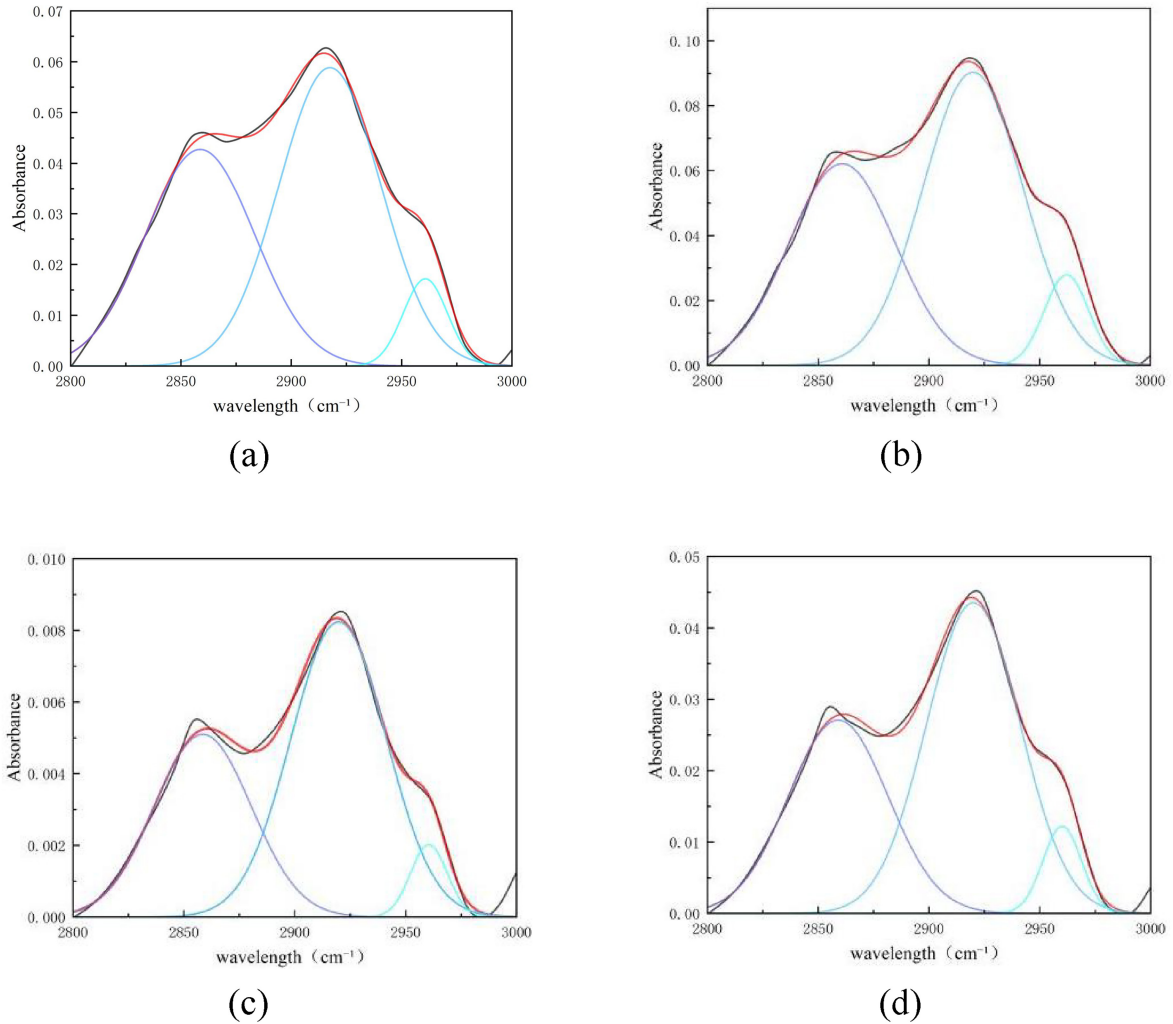
Peak fitting of the two types of coal in the aliphatic region before and after desulfurization. **(a)** Fitting results of Coal 092a before desulfurization. **(b)** Fitting results of Coal 092a after desulfurization. **(c)** Fitting results of Coal 100b before desulfurization. **(d)** Fitting results of Coal 100b after desulfurization.

**FIGURE 13:**
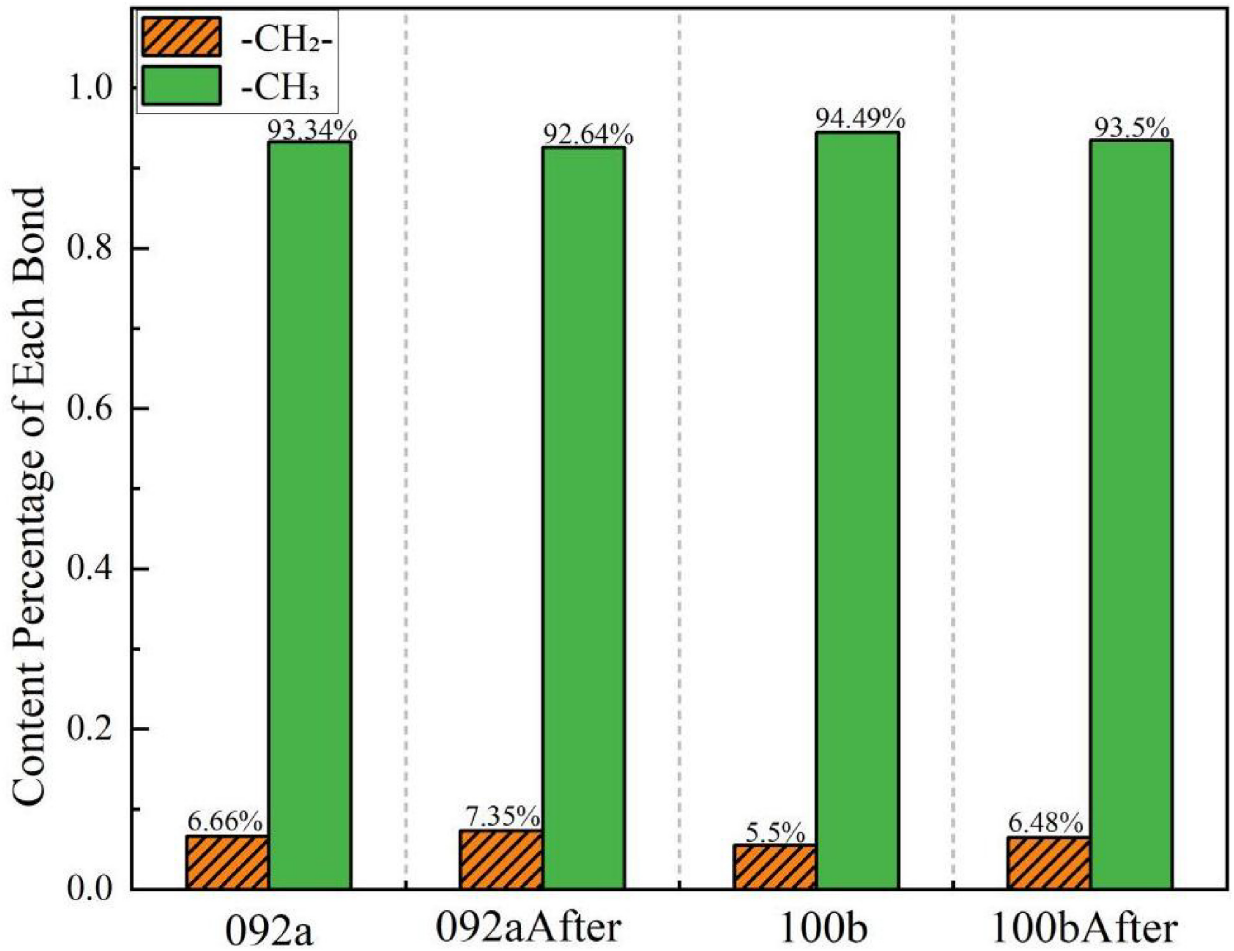
Content proportion of each component in the aliphatic region.

Figure 12 shows the peak fitting results of the two types of coal in the aliphatic region (2,800–3,000 cm^–1^) before and after desulfurization. The content proportion of each component in this region was calculated based on the fitted peak area in Figure 12, as presented in Figure 13.

The relative content of components representing methylene in both coal samples increased, while that representing methyl groups decreased correspondingly. This indicates that Pseudomonas putida exhibits selectivity for aliphatic structures during its metabolic process, preferentially utilizing methyl groups located at the molecular terminals as carbon and energy sources (Wang et al., 2024). In contrast, it has a weaker ability to degrade methylene structures inside long chains, ultimately leading to methylene enrichment.

The change trends in the aliphatic region of the two coal samples are consistent, but the magnitude of change is determined by the initial content of aliphatic chains in the coal matrix. Coal 100b has a more open molecular structure of the coal matrix, and its aliphatic component content is significantly higher than that of Coal 092a. The more abundant methyl groups provide sufficient metabolic substrates for microorganisms, leading to the extensive consumption of methyl groups. Meanwhile, the relative enrichment effect of methylene—due to its strong degradation resistance—becomes more prominent. This is related to the more active microbial metabolism during its efficient desulfurization process. In contrast, most of the aliphatic chains in Coal 092a are embedded in highly condensed aromatic condensed rings. The total amount of aliphatic side chains is small and encapsulated by aromatic structures, resulting in limited methyl substrates accessible to microorganisms. Thus, the decrease in methyl groups is smaller, and the degree of relative methylene enrichment is also weaker than that in Coal 100b. This is consistent with the low desulfurization rate of only 17.6% for this coal sample.

In summary, the abundance of aliphatic chains in the coal matrix is the core prerequisite for the selective degradation by microorganisms. Coal matrices with high aliphatic chain content and good exposure are more prone to selective evolution of aliphatic structures, while those with scarce aliphatic chains and high encapsulation degree exhibit more moderate changes in functional groups.

#### 3.3.3 Peak fitting of the oxygen-containing functional group region

**FIGURE 14:**
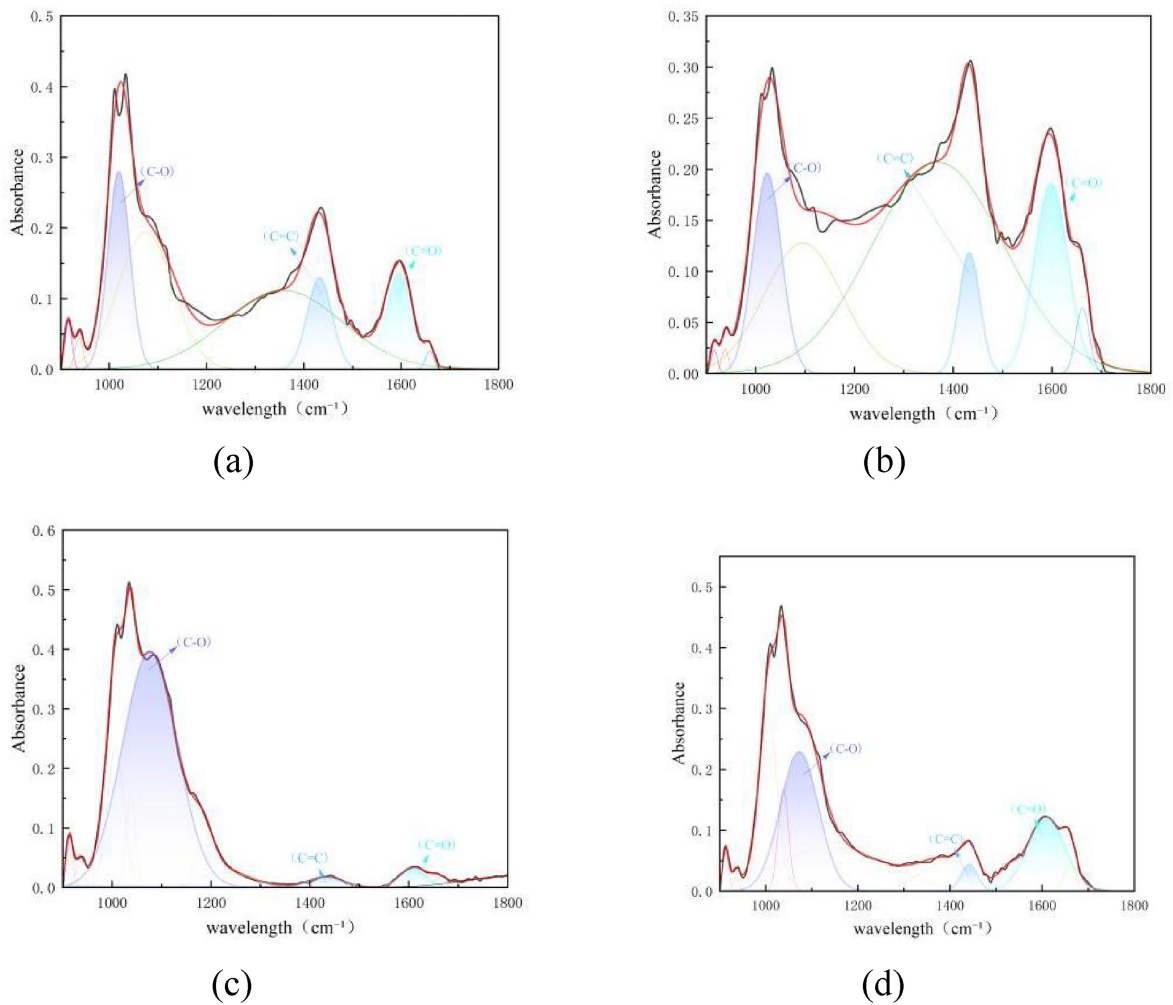
Peak fitting of the two types of coal in the aliphatic region before and after desulfurization. **(a)** Fitting results of Coal 092a before desulfurization. **(b)** Fitting results of Coal 092a after desulfurization. **(c)** Fitting results of Coal 100b before desulfurization. **(d)** Fitting results of Coal 100b after desulfurization.

**FIGURE 15:**
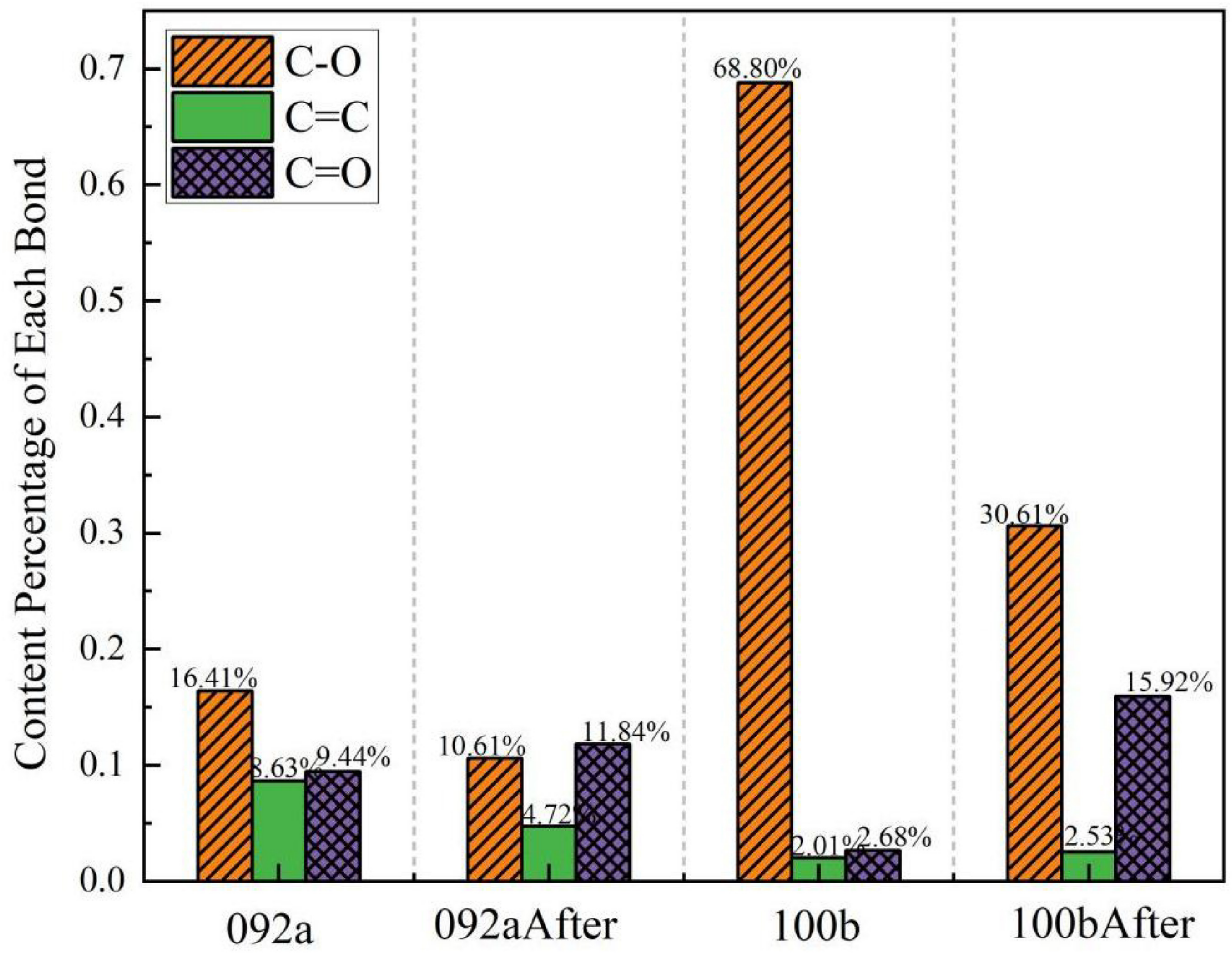
Content proportion of each component in the oxygen-containing functional group region.

Figure 14 shows the peak fitting results of the two types of coal in the oxygen-containing functional group region (900–1,800 cm^–1^) before and after desulfurization. The content proportion of each component in this region was calculated based on the fitted peak area in Figure 14, as presented in Figure 15. Results show that for Coal 092a, the C-O content in the oxygen-containing functional group region decreased from 16.41 to 10.61%, the C = C bond content dropped from 8.63 to 4.72%, and the C = O bond content increased from 9.44 to 11.84%. For Coal 100b, the C-O content in this region decreased from 60.8 to 30.61%, the C = C bond content rose from 2.01 to 2.53%, and the C = O bond content increased from 2.68 to 15.92%.

For Coal 092a, the C = O bond content shows a significant increase. This change directly indicates that intense oxidation reactions took place during the desulfurization process. After aliphatic or aromatic structures in the coal matrix are oxidized, a large number of oxygen-containing functional groups are generated. These groups mainly include carbonyl groups (C = O), carboxyl groups (-COOH), aldehyde groups (-CHO), and keto groups (-C = O), all of which contribute to the obvious rise in C = O bond proportion. The proportion of C-O bonds displays a notable decline, which demonstrates the cleavage of key functional groups in coal, such as ether bonds (C-O-C) and phenolic hydroxyl groups (C-OH). This bond breakage is caused by oxidation reactions, as oxidizing substances attack and disrupt these relatively unstable chemical linkages. Combined with the increase in C = O bonds, the decrease in C = C bond proportion allows us to infer a further structural change. Some aromatic rings are oxidized and undergo ring-opening reactions, and these damaged aromatic structures are then converted into aliphatic carboxylic acids or ketone structures. During the desulfurization process, microorganisms produce oxidizing substances. These substances preferentially oxidize the coal macromolecular structure, leading to the formation of a large number of C = O groups. At the same time, oxidation reactions can also trigger cross-linking reactions between coal macromolecules. This cross-linking effect makes the overall coal structure more condensed and dense. This process consumes a portion of small-molecule aromatic rings and C-O bonds, which further reduces the relative proportions of C = C and C-O. In contrast, the more stable C = O bonds and large condensed ring structures show an increase in their relative proportions.

For Coal 100b, the proportion of C = O bonds increases dramatically, and the growth range is far larger than that of Coal 092a. This result proves that Coal 100b experienced more intense oxidation reactions, which also reaffirms that the treatment process applied to Coal 100b was more vigorous. The proportion of C = C bonds rises slightly, but this change is not caused by the formation of new aromatic rings. Instead, it is a passive relative increase. After the C-O bonds in the aliphatic regions of Coal 100b are extensively consumed, the relative proportion of the originally extremely low aromatic core C = C bonds goes up passively. Microorganisms secrete strong oxidases, which non-selectively attack the oxygen-rich macromolecular network of Coal 100b. These oxidases preferentially cleave the C-O ether bonds that connect different structural units in the coal. As a result, the entire molecular network depolymerizes into a large number of small-molecule fragments. These small fragments are further oxidized, generating a large quantity of carboxylic acids and ketones containing C = O functional groups. This series of reactions ultimately leads to the characteristic sharp surge in the proportion of C = O bonds.

The difference in functional group changes between the two coal samples is essentially rooted in the differences in the initial ether bond density and molecular network structure of the coal matrix. Ether bonds (C-O-C) are the key bridging bonds that connect the structural units of coal macromolecules. Their density directly determines the looseness of the coal matrix and the accessibility of microbial enzymes to the internal structure. Coal 100b has a higher ether bond density than Coal 092a. Its molecular network is mainly composed of aromatic rings and sulfone structures, and it lacks cross-linking of high-density aromatic condensed rings. As a result, the ether bonds, which act as the main connecting units, are highly exposed and easy to target. The oxidases secreted by Pseudomonas putida can efficiently cleave these exposed ether bonds, causing the C-O bond content to drop sharply from 60.8 to 30.61%. The small-molecule fragments produced by ether bond cleavage are further oxidized, generating a large number of C = O-containing functional groups. This ultimately leads to a significant surge in the C = O proportion, from 2.68 to 15.92%. In contrast, the matrix of Coal 092a is dominated by highly condensed aromatic rings. It has a low content of ether bonds, and most of these ether bonds are encapsulated by dense aromatic structures. This makes it difficult for microbial enzymes to access and catalyze their cleavage. Therefore, the C-O bond content only decreases slightly from 16.41 to 10.61%. The limited cleavage products generated from ether bond breakage are not enough to support the massive formation of C = O groups, so only mild oxidation reactions occur in Coal 092a. Meanwhile, its high aromaticity leads to tight cross-linking between macromolecules. The oxidation process is accompanied by partial structural condensation, which inhibits drastic changes in the types and proportions of functional groups.

In summary, the ether bond density of the coal matrix directly determines the microbial depolymerization efficiency of coal macromolecules. Loose matrices with high ether bond density (Coal 100b) are prone to ether bond cleavage and oxidative upgrading, while dense matrices with scarce ether bonds (Coal 092a) exhibit the characteristics of “weak depolymerization and weak oxidation,” ultimately resulting in a significant difference in their desulfurization efficiencies.

#### 3.3.4 Peak fitting of the aromatic hydrocarbon region

**FIGURE 16:**
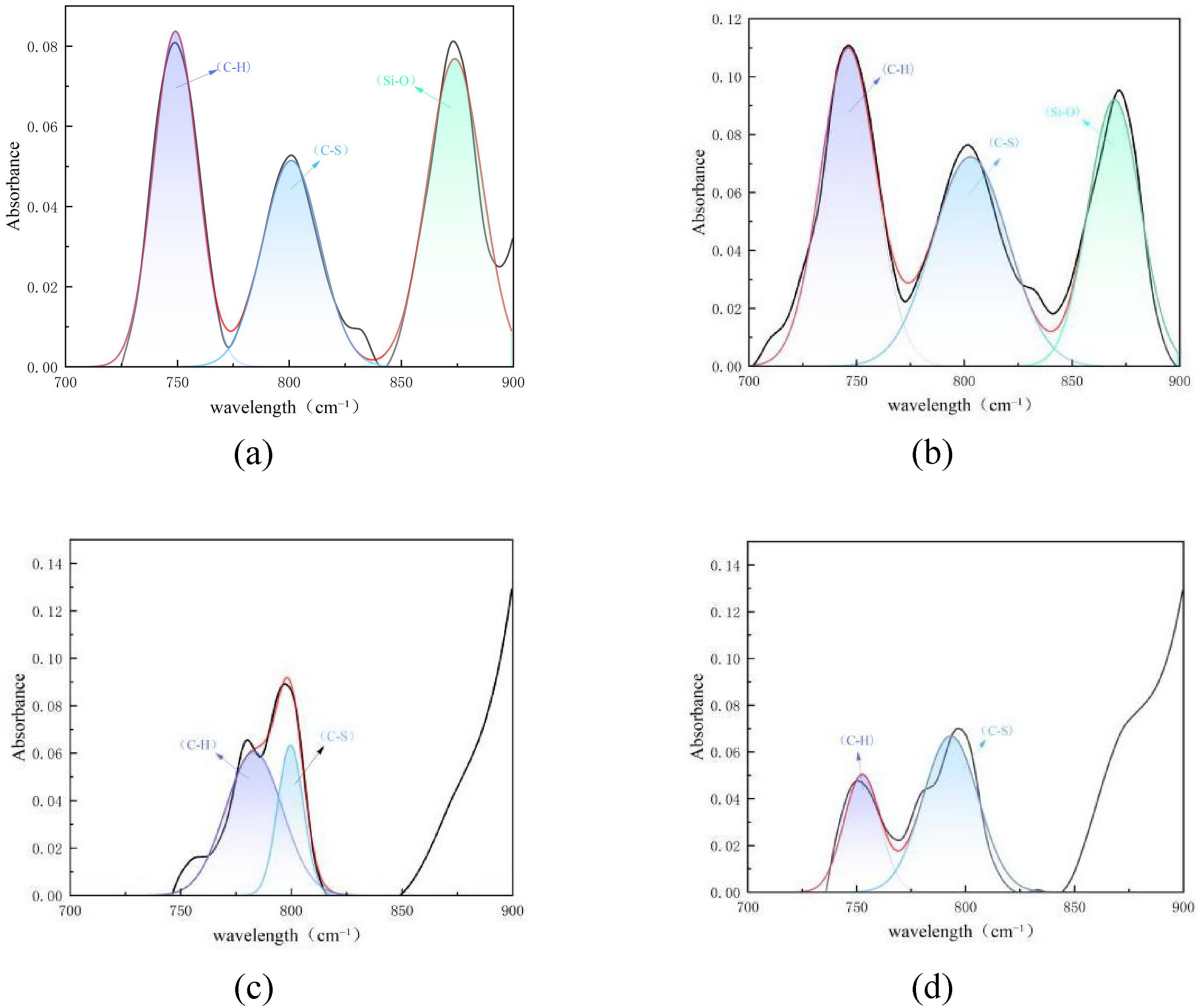
Peak fitting of the two types of coal in the aromatic hydrocarbon region before and after desulfurization. **(a)** Fitting results of Coal 092a before desulfurization. **(b)** Fitting results of Coal 092a after desulfurization. **(c)** Fitting results of Coal 100b before desulfurization. **(d)** Fitting results of Coal 100b after desulfurization.

**FIGURE 17:**
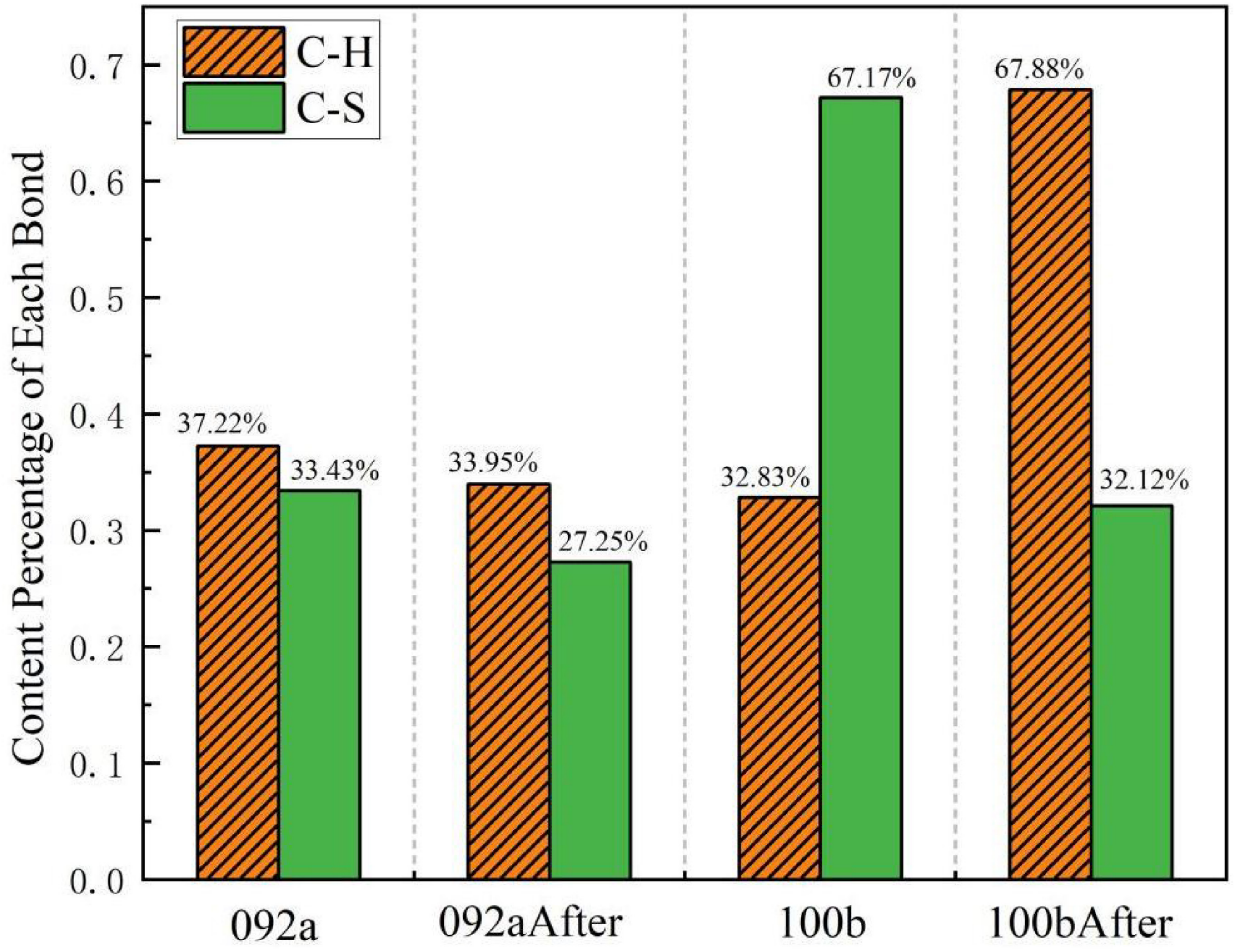
Content proportion of each component in the aromatic hydrocarbon region.

Figure 16 shows the peak fitting results of the two types of coal in the aromatic hydrocarbon region (700–900 cm^–1^) before and after desulfurization. The content proportion of each component in this region was calculated based on the fitted peak area in Figure 16, as presented in Figure 17. Results indicate that for Coal 092a, the fitting peak area of C-H bonds decreased from 37.22 to 33.95%, and that of C-S bonds dropped from 33.43 to 27.25%. For Coal 100b, the fitting peak area of C-H bonds increased from 32.83 to 67.88%, while that of C-S bonds decreased from 67.17 to 32.12%.

The significant differences in the aromatic hydrocarbon region of the two coal samples essentially stem from the varying encapsulation degrees of sulfur-containing functional groups by the aromatic rings in the coal matrix. This directly affects the contact efficiency between microbial enzymes and C-S bonds, thereby leading to the aforementioned differences in desulfurization rates. Coal 092a has an extremely high aromaticity and condensation degree, with its organic sulfur embedded inside aromatic condensed rings, forming strong steric hindrance. This structure makes it difficult for the C-S bond-cleaving enzymes secreted by microorganisms to access the substrates, only allowing the cleavage of a small number of C-S bonds exposed at the edges of the condensed rings. Consequently, the proportion of C-S bonds only shows a slight decrease. Meanwhile, the high encapsulation degree protects the aromatic ring C-H structures, resulting in only a minor reduction in their proportion due to mild oxidative condensation. In contrast, Coal 100b has a lower aromatic ring encapsulation degree. Its organic sulfur is mainly composed of sulfones and thiophenes, and the aromatic rings are mostly small clusters without encapsulation by high-density condensed rings, leading to good exposure of sulfur-containing functional groups. To obtain energy, microorganisms can secrete non-specific extracellular enzymes to first cleave the bridging bonds connecting aromatic clusters, causing the depolymerization of the macromolecular network. This exposes the previously hidden C-S bonds, which are then extensively cleaved, resulting in a decrease in the proportion of C-S bonds. Simultaneously, the cleavage of bridging bonds disperses the aromatic ring clusters, exposing the previously masked aromatic ring C-H. Coupled with the extensive degradation of aliphatic structures, the relative proportion of C-H bonds ultimately surged from 32.83 to 67.88%. To acquire energy, microorganisms not only cleave C-S bonds but also secrete enzymes that extensively attack the coal’s macromolecular network, cutting the bridging bonds between aromatic clusters and thus breaking down large structures into small ones (Tsugawa et al., 2019). The generated small-molecule fragments are absorbed and utilized by microorganisms as carbon and energy sources, further promoting the degradation process.

In summary, the encapsulation degree of aromatic rings in the coal matrix is the key limiting factor for C-S bond cleavage efficiency. Loose matrices with low encapsulation degree enable efficient desulfurization and exposure of aromatic structures, while dense matrices with high encapsulation degree seriously hinder microbial removal of organic sulfur and modification of aromatic structures. This mechanism forms a complete logical closed loop with the differences in desulfurization rates of the two coal samples in the previous morphological sulfur analysis, clarifying the core influencing role of coal matrix structure on microbial desulfurization efficiency.

## 4 Mechanism analysis of microbial desulfurization

**FIGURE 18:**
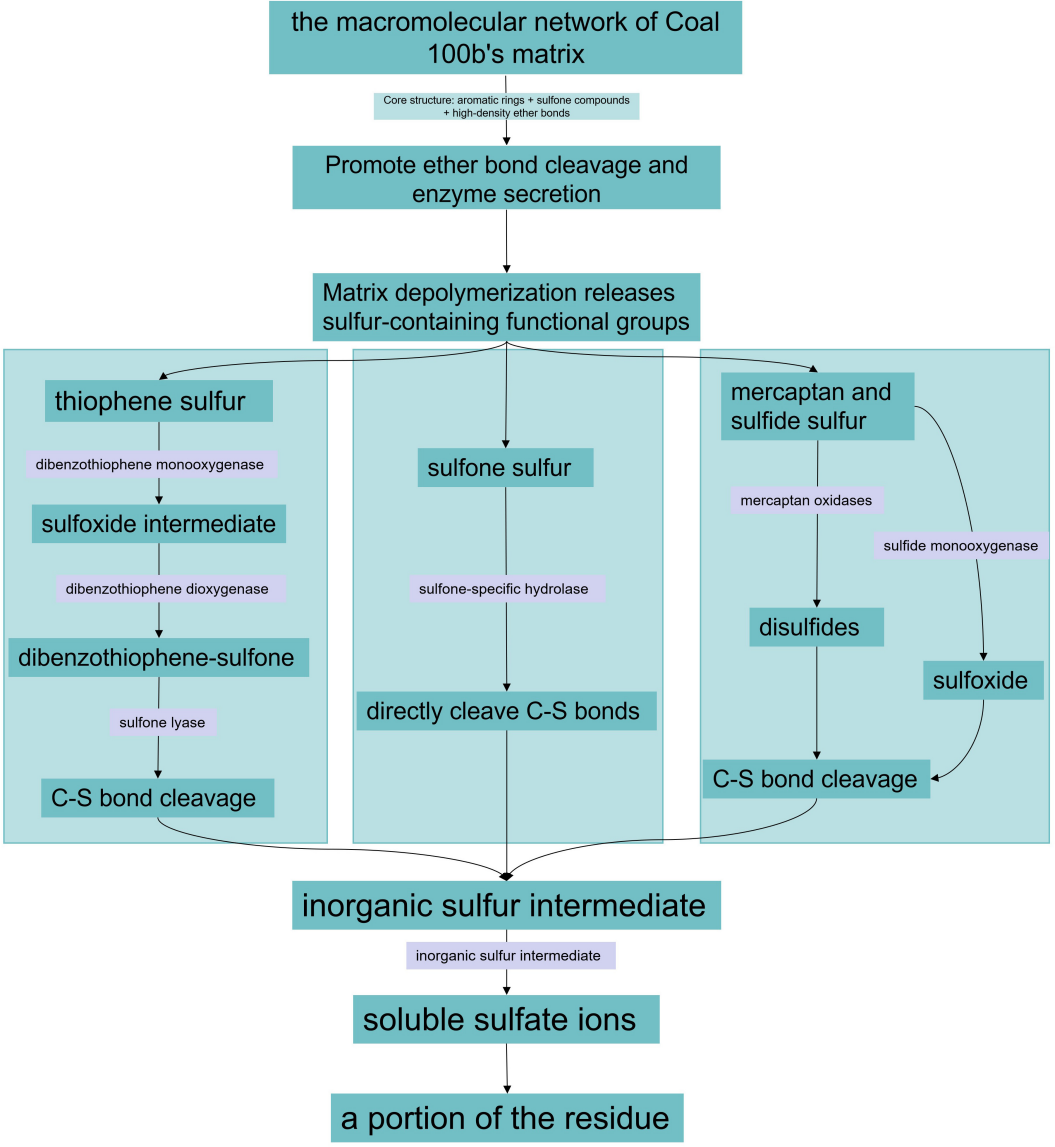
Schematic diagram of the desulfurization pathway.

By conducting desulfurization experiments on the two coal samples, it was found that there were significant differences in their desulfurization rates. To clarify the core mechanism of efficient desulfurization and reveal the key influencing factors, the mechanism analysis was focused on Coal 100b with a higher desulfurization rate. Research shows that the desulfurization pathway of Coal 100b is centered on the synergistic effect of specific cleavage of C-S bonds and sulfur oxidation, accompanied by the cometabolic process of coal matrix macromolecule depolymerization, ultimately achieving efficient removal of organic sulfur. Figure 18 shows the desulfurization pathway of Coal 100b.

The coal matrix of Coal 100b takes aromatic rings and sulfone structures as core units, forming a loose macromolecular network connected by high-density ether bonds. This structural feature provides advantages for microbial desulfurization. Pseudomonas putida first secretes non-specific oxidases to attack the ether bond bridging structures in the coal matrix, causing the C-O bond content to plummet from 60.8 to 30.61%. The cleavage of ether bonds induces the depolymerization of the originally cross-linked macromolecular network, releasing sulfur-containing functional groups such as thiophenes, mercaptans, sulfides, and sulfones embedded within it from spatial encapsulation. This significantly improves the contact efficiency between microbial enzyme systems and substrates.

Thiophene sulfur accounts for a significant proportion in Coal 100b, but since the aromatic rings exist as small clusters and do not form high-density condensed ring shielding, this provides conditions for the initiation of desulfurization. As can be seen from the decreased peak area of sulfoxides in XPS and the appearance of the characteristic vibration peak of DBTO2 at 832 cm^–1^ in FTIR mentioned above, the dibenzothiophene monooxygenase secreted by Pseudomonas putida first attacks the thiophene ring, oxidizing it to a sulfoxide intermediate, which is then further oxidized to sulfone products under the action of dibenzothiophene dioxygenase. Finally, sulfone lyase specifically cleaves the C-S bonds in the sulfone structure, causing sulfur atoms to detach from the aromatic ring skeleton. This process directly leads to a simultaneous decrease in the fitting peak areas of thiophenes, sulfoxides, and sulfones in XPS, and the proportion of C-S bonds in the aromatic hydrocarbon region plummets from 67.17 to 32.12%. The desulfurization pathway of thiophene-based organic sulfur conforms to the 4S (Test Sieves, 2000; National Technical Committee of Coal Standardization, 2003) desulfurization pathway. Figure 18a shows the desulfurization pathway of thiophene-based sulfur.

Coal 100b has a high initial proportion of sulfone sulfur with good exposure, and Pseudomonas putida exhibits efficient catalytic activity toward it. The sulfone-specific hydrolase secreted by the strain can act directly on the R-SO_2_-R structure, cleaving C-S bonds without multi-step oxidation. The released sulfur fragments are converted to sulfite by extracellular oxidases, and finally oxidized to sulfate. This process is also reflected in the increase of sulfate sulfur from 0.08 to 0.38%. The desulfurization pathway of sulfone sulfur forms a synergy with the 4S pathway (Li et al., 2008; Wang et al., 2003) of thiophenes, jointly improving the organic sulfur removal efficiency, resulting in an organic sulfur removal rate of 60.9% for Coal 100b. Figure 18b shows the desulfurization pathway of sulfone sulfur.

For mercaptan and sulfide-based organic sulfur, they have lower C-S bond energy and higher chemical activity. Under the action of mercaptan oxidases and sulfide monooxygenases secreted by Pseudomonas putida, mercaptans are first oxidized to disulfides, while sulfides are oxidized to sulfoxides. Both are subsequently converted to inorganic sulfur intermediates through C-S bond cleavage, and finally form sulfate ions. Figure 18c shows the desulfurization pathway of mercaptan and sulfide-based sulfur. Due to the low initial content of mercaptans and sulfides in Coal 100b, their degradation contributes less to the total desulfurization efficiency than thiophenes and sulfones, but they are still an important part of the desulfurization pathway.

The sulfur atoms and inorganic sulfur intermediates released by C-S bond cleavage are ultimately converted to soluble sulfate ions under the action of the sulfur oxidase system of Pseudomonas putida. A portion of the sulfate remains on the coal sample surface, resulting in a significant increase in the detected sulfate sulfur content. The rest dissolves in the reaction solution and is removed by subsequent washing, achieving effective removal of sulfur from coal.

In summary, the efficient desulfurization of Coal 100b benefits from the precise matching between its matrix characteristics and the microbial enzyme system, and the desulfurization pathway exhibits three core characteristics. Firstly, matrix depolymerization dominated by ether bond cleavage provides a prerequisite for the exposure of sulfur-containing functional groups, serving as the foundation for high desulfurization efficiency. Secondly, centered on the 4S metabolic pathway, it achieves hierarchical oxidation and specific desulfurization of various organic sulfur species such as thiophenes and sulfones. Finally, the utilization of aliphatic carbon sources and the matrix oxidation process provide energy for the desulfurization reaction, forming a positive feedback of desulfurization metabolism. This pathway fully reflects the deterministic role of coal matrix characteristics in the microbial desulfurization mechanism, laying an important theoretical foundation for the precise and targeted application of efficient coal biodesulfurization technology.

## 5 Conclusion

(1) Results of morphological sulfur analysis showed that there were significant differences in desulfurization efficiency of Pseudomonas putida between the two coal samples. The organic sulfur removal rate of Coal 100b reached 60.9%, which was much higher than that of Coal 092a (17.6%). The sulfate sulfur content of both coal samples increased significantly, confirming that microorganisms converted sulfur into sulfate ions through oxidation, with a portion remaining on the coal sample surface. The increase in pyritic sulfur content was a change in relative proportion: pyrite has a stable structure and is difficult to degrade, while the massive removal of organic sulfur reduced the total sulfur content of the coal samples. In summary, the main removal target of the strain is organic sulfur, and it has limited ability to remove pyritic sulfur. Coal matrix characteristics are the key factor determining the microbial desulfurization effect. In this study, only two coal samples with significantly different structures from the Datong mining area were selected as research objects, without covering coal samples of different ranks and origins, so the universality of the relevant conclusions still needs further verification.
(2) Results of XPS and FTIR analyses indicated that Pseudomonas putida exhibited significant differences in desulfurization efficiency between Coal 092a and Coal 100b, with the core depending on coal matrix characteristics and sulfur occurrence forms. XPS results showed that the strain efficiently removed sulfone-type sulfur from both coals: Coal 100b could degrade various organic sulfur species such as thiophenes and sulfides, while Coal 092a was only effective for sulfones. FTIR peak-fitting results revealed that Coal 100b had a loose structure, abundant ether bonds, and good sulfur exposurecurrence forms. XPS results showed that the strain efficiently removed further verification.pyrite has a sfor the precdegree seriously hinder microbia-S bonds cleaved. In contrast, Coal 092a had high aromaticity and a dense structure, with sulfur-containing functional groups embedded in condensed rings and large steric hindrance, leading to gentle changes in various functional groups. In summary, the original chemical structure of coal is the key factor determining microbial desulfurization efficiency. In this study, the desulfurization mechanism was only analyzed from the macroscopic functional group level, and the binding sites between enzymes and sulfur-containing functional groups in coal matrix were not explored at the molecular level. In the future, microscopic mechanism research can be carried out combined with technologies such as molecular simulation and proteomics.
(3) The efficient desulfurization of Coal 100b follows a synergistic mechanism of matrix depolymerization, hierarchical oxidation, and C-S bond cleavage, with the 4S metabolic pathway as the core desulfurization route. Pseudomonas putida first cleaves ether bonds in the coal matrix via non-specific oxidases, achieving macromolecular network depolymerization and exposure of sulfur-containing functional groups. Then, through specific enzyme systems such as dibenzothiophene monooxygenase and sulfone lyase, it completes the 4S metabolic pathway degradation of thiophene-based sulfur and direct catalytic cleavage of sulfone sulfur, while synergistically degrading mercaptans and sulfides. Finally, organic sulfur is converted into soluble sulfate ions.

## Data Availability

The data presented in the study are deposited in the NCBI Nucleotide Repository, accession number FJ477106.

## References

[B1] Akimbekov N. S. Digel I. Tastambek K. T. Marat A. K. Turaliyeva M. A. Kaiyrmanova G. K. (2022). Biotechnology of microorganisms from coal environments: From environmental remediation to energy production. *Biology* 11:1306. 10.3390/biology11091306 36138784 PMC9495453

[B2] Akimbekov N. Digel I. Abdieva G. Ualieva P. Tastambek K. (2021). Lignite biosolubilization and bioconversion by *Bacillus sp*.: The collation of analytical data. *Biofuels* 12 247–258. 10.1080/17597269.2020.1753936

[B3] Altynbay N. Tastambek K. Akimbekov N. Digel I. Tagayev K. Kamenov B. et al. (2024). Comprehensive review on enhancing saline soil reclamation efficiency through bacterial solubilization of low-rank coal. *Eng. Sci.* 30:1147. 10.30919/es1147

[B4] Aytar P. Kay M. C. Mutlu B. M. Çabuk A. (2013). Coal desulfurization with *Acidithiobacillus ferrivorans*, from balya acidic mine drainage. *Energy Fuels* 27 3090–3098. 10.1021/ef400360t

[B5] Channa H. F. Khoso A. S. Soomro A. S. Uqaili M. A. (2025). Studies on sulfur liberation characteristics of Pakistani high sulfur low-rank coal using *Hardgrove grinding*. *Intern. J. Coal Preparat. Util.* 45 548–567. 10.1080/19392699.2024.2341953

[B6] Cheng G. Peng Y. J. Duan P. G. Li E. Z. Lv C. Wang X. (2025). Sustainable desulfurization of fine high-sulfur coal via flotation-electrochemical Method[J/OL]. *Minerals Eng.* 233:109571. 10.1016/j.mineng.2025.109571

[B7] Dong X. W. Li X. Q. Wang F. S. Han T. S. Dong X. M. (2024). Study on the inhibition of spontaneous combustion characteristics of high-sulfur coal by *Gloeophyllum trabeum*. *J. China Coal Soc.* 49 1052–1067. 10.13225/j.cnki.jccs.2024.0388

[B8] Guan X. Y. Wang B. Jiang J. W. Tian J. S. Dong Y. Chen Z. et al. (2020). Comparative study on sediment microbial communities in *Fenneropenaeus chinensis* polyculture ponds. *Mar. Sci. Bull.* 39 730–739.

[B9] Gupta N. Roychoudhury K. P. Deb K. J. (2004). Biotechnology of desulfurization of diesel: Prospects and challenges. *Appl. Microbiol. Biotechnol.* 66 356–366. 10.1007/s00253-004-1755-7 15538557

[B10] Handayani I. Paisal Y. Soepriyanto S. Chaerun S. K. (2016). Biodesulfurization of organic sulfur in Tondongkura coal from Indonesia by multi-stage bioprocess treatments. *Hydrometallurgy* 16 884–893. 10.1016/j.hydromet.2016.10.027

[B11] Li S. S. Li G. Q. Ma T. Liang F. L. Liu R. L. (2008). Functional correlation between benzothiophene and dibenzothiophene desulfidase. *Environ. Sci.* 11, 3166–3171. 10.13227/j.hjkx.2008.11.001 19186822

[B12] Liu F. Cao W. J. Zhang J. M. Cao G. M. Guo L. F. (2021). Progress of scientific and technological innovation in China’s coal industry and the development direction of the “14th Five-Year Plan”. *J. China Coal Soc.* 46 1–15. 10.13225/j.cnki.jccs.2021.0042

[B13] Liu J. Yang S. W. Zhao W. Hu B. Xia Y. G. Ma S. W. et al. (2022). Research progress on the migration and transformation mechanism of thiophene sulfides in coal pyrolysis. *J. China Coal Soc.* 47 3886–3896. 10.13225/j.cnki.jccs.lc22.1030

[B14] Marinov S. Gonsalvesh L. Stefanova M. Yperman J. Carleer R. Reggers G. et al. (2009). Combustion behaviour of some biodesulphurized coals assessed by TGA/DTA. *Thermochim. Acta* 497 46–51. 10.1016/j.tca.2009.08.012

[B15] Mohebali G. Ball S. A. (2016). Biodesulfurization of diesel fuels – Past, present and future perspectives. *Intern. Biodeteriorat. Biodegradat.* 110 163–180. 10.1016/j.ibiod.2016.03.011

[B16] National Technical Committee of Coal Standardization (2003). *Determination Methods for Various Forms of Sulfur in Coal: GB/T 215-2003.* Beijing: China Standards Press.

[B17] Oluwafolakemi O. S. Olawale M. D. (2023). Bio-catalytic degradation of dibenzothiophene (DBT) in petroleum distillate (diesel) by *Pseudomonas spp*. *Sci. Rep.* 13:6020. 10.1038/s41598-023-31951-8 37055435 PMC10102322

[B18] Pokorný R. Olejníková P. Balog M. Zifčák P. Hölker U. Janssen M. et al. (2005). Characterization of microorganisms isolated from lignite excavated from the Záhorie coal mine (southwestern Slovakia). *Res. Microbiol.* 156 932–943. 10.1016/j.resmic.2005.04.010 16085397

[B19] Shi B. (2011). Pre-combustion desulfurization and enhanced desulfurization methods of high-sulfur coal. *Coal Preparat. Technol.* 68–71. 10.16447/j.cnki.cpt.2011.02.027

[B20] Soleimani M. Bassi A. Margaritis A. (2007). Biodesulfurization of refractory organic sulfur compounds in fossil fuels. *Biotechnol. Adv.* 25 570–596. 10.1016/j.biotechadv.2007.07.003 17716849

[B21] Test Sieves. (2000). Technical Requirements and Testing — Part 1: Test Sieves of Wire Cloth: ISO 3310-1-2000. Geneva: ISO.

[B22] Tong M. Y. Ma T. Zhang Q. Liang F. L. Liu R. L. (2005). Selective removal of organic sulfur from fuel oil by resting cell method. *Environ. Sci.* 24–27. 10.13227/j.hjkx.2005.01.006 15861535

[B23] Tsugawa S. Noda Y. Tarumi R. Mimura Y. Yoshida K. Iwata Y. et al. (2019). Glutathione levels and activities of glutathione metabolism enzymes in patients with schizophrenia: A systematic review and meta-analysis. *J. Psychopharmacol.* 33 1199–1214. 10.1177/0269881119845820 31039654

[B24] Wang F. Yao X. Z. Yang Y. Zhao Y. Dong W. Y. Wang H. J. et al. (2024). Effects of salinity on cometabolic degradation of polycyclic aromatic hydrocarbons in sediment and microbial community response. *J. Harbin Instit. Technol.* 56 161–170.

[B25] Wang R. Liu Z. Y. Wang Q. Peng C. N. Chen Z. H. Li F. L. et al. (2021). Experimental on CO_2_-assisted aerobic-bacteria coal desulfurization process. *Chem. Indus. Eng. Prog.* 40 526–533. 10.16085/j.issn.1000-6613.2020-0416

[B26] Wang X. Y. Zhang Y. K. Liang B. (2003). Research on microbial desulfurization process conditions. *Environ. Sci.* 44–48. 10.13227/j.hjkx.2003.05.008

[B27] Xia J. Qin M. L. Gao F. Shan Y. F. Mu X. G. Sun R. J. et al. (2022). Effect of bromine water desulfurization on the characteristic temperature and active groups of coal spontaneous combustion. *J. Coal Convers.* 45 29–42. 10.19726/j.cnki.ebcc.202201004

[B28] Zhang D. C. Zhang M. X. Chen Q. R. Li Q. (2005). Research on the magnetization cultivation of coal desulfurization bacteria and the mechanism of magnetobiological effects. *J. China Coal Soc.* 484–488. 10.13225/j.cnki.jccs.2005.04.017

[B29] Zhang D. M. Xie Q. L. Zhang P. Zhang C. F. (2008). Experimental study on coal desulfurization by white-rot fungi. *Energy Technol. Manag.* 67–69.

[B30] Zhao Y. Wang X. H. Xu M. N. Wang T. H. (2018). Research progress on high-sulfur coal desulfurization. *Technol. Dev. Chem. Indus.* 47 26–28. 10.3389/fchem.2025.1698815 41322379 PMC12660220

