# Supplementary material for: Study on the effects and mechanisms of different coal matrix structures on the desulfurization efficiency of Pseudomonas putida
Source: Front Microbiol. 2026 Apr 15;17:1820915. doi: 10.3389/fmicb.2026.1820915 (PMC13125091; doi:10.3389/fmicb.2026.1820915)
Supplement: Supplementary file 1 [file Table_1.doc]

实验菌dna

TGACGGGAGCTTGCTCCTTGATTCAGCGGCGGACGGGTGAGTAATGCCTAGGAATCTGCCTGGTAGTGGGGGACAACGTT

TCGAAAGGAACGCTAATACCGCATACGTCCTACGGGAGAAAGCAGGGGACCTTCGGGCCTTGCGCTATCAGATGAGCCTA

GGTCGGATTAGCTAGTTGGTGGGGTAATGGCTCACCAAGGCGACGATCCGTAACTGGTCTGAGAGGATGATCAGTCACAC

TGGAACTGAGACACGGTCCAGACTCCTACGGGAGGCAGCAGTGGGGAATATTGGACAATGGGCGAAAGCCTGATCCAGC

CATGCCGCGTGTGTGAAGAAGGTCTTCGGATTGTAAAGCACTTTAAGTTGGGAGGAAGGGCAGTAAGTTAATACCTTGCTG

TTTTGACGTTACCGACAGAATAAGCACCGGCTAACTCTGTGCCAGCAGCCGCGGTAATACAGAGGGTGCAAGCGTTAATCG

GAATTACTGGGCGTAAAGCGCGCGTAGGTGGTTTGTTAAGTTGGATGTGAAAGCCCCGGGCTCAACCTGGGAACTGCATCC

AAAACTGGCAAGCTAGAGTACGGTAGAGGGTGGTGGAATTTCCTGTGTAGCGGTGAAATGCGTAGATATAGGAAGGAACA

CCAGTGGCGAAGGCGACCACCTGGACTGATACTGACACTGAGGTGCGAAAGCGTGGGGAGCAAACAGGGATTAGATACCC

TGGTAGTCCACGCCGTAAACGATGTCAACTAGCCGTTGGAATCCTTGAGATTTTAGTGGCGCAGCTAACGCATTAAGTTGAC

CGCCTGGGGAGTACGGCCGCAAGGTTAAAACTCAAATGAATTGACGGGGGCCCGCACAAGCGGTGGAGCATGTGGTTTAAT

TCGAAGCAACGCGAAGAACCTTACCAGGCCTTGACATGCAGAGAACTTTCCAGAGATGGATTGGTGCCTTCGGGAACTCTG

ACACAGGTGCTGCATGGCTGTCGTCAGCTCGTGTCGTGAGATGTTGGGTTAAGTCCCGTAACGAGCGCAACCCTTGTCCTTA

GTTACCAGCACGTTATGGTGGGCACTCTAAGGAGACTGCCGGTGACAAACCGGAGGAAGGTGGGGATGACGTCAAGTCATC

ATGGCCCTTACGGCCTGGGCTACACACGTGCTACCAATGGTCGGTACAGAGGGTTGCCAAGCCGCGAGGTGGAGCTAATCTC

ACAAAACCGATCGTAGTCCGGATCGCAGTCTGCAACTCGACTGCGTGAAGTCGGAATCGCTAGTAATCGCGAATCAGAATG

TCGCGGTGAATACGTTCCCGGGCCTTGTACACACCGCCCGTCACACCATGGGAGTGGGTTGCACCAGAAGTAGC
